# Heart disease prediction using rough neutrosophic sets and dual-attention neural networks: RNS-OptiDANet

**DOI:** 10.3389/frai.2026.1792860

**Published:** 2026-04-13

**Authors:** T. Ashika, G. Hannah Grace

**Affiliations:** Department of Mathematics, School of Advanced Sciences, Vellore Institute of Technology, Chennai, India

**Keywords:** attention mechanism, machine learning, neural network, rough neutrosophic sets, rough set theory, uncertainty handling

## Abstract

**Introduction:**

Heart disease is a major global health problem that highlights the need for effective and accurate prediction methods.

**Methods:**

This paper presents RNS-OptiDANet, a hybrid framework that combines rough set theory (RST), rough neutrosophic sets (RNS) and an optimized dual-attention neural network (OptiDANet) in order to predict heart disease. For feature selection, the QuickReduct method with the discernibility matrix (RST QRDM) was used. The features selected in RST were represented as RNS representations to deal with uncertainty in the classification process. The OptiDANet model implements Dual Attention Mechanisms such as Channel Attention (CAM) and Soft Attention Mechanism (SAM) to highlight the relevant patterns while reducing noise. The performance of the developed framework has been improved through Hyperparameter tuning using Optuna and overfitting has been avoided. Finally, classification is conducted using a Random Forest (RF) model.

**Results:**

Experimental results demonstrate strong performance in terms of accuracy, precision, recall and F1-score across datasets.

**Discussion:**

An eXplainable Artificial Intelligence (XAI) module is integrated to provide feature level interpretability and clinical transparency while ablation study validates the contribution of each framework component confirming the robustness and effectiveness of the proposed hybrid RNS-OptiDANet model.

## 1 Introduction

Cardiovascular disease (CVD) ranks as a leading cause of death responsible for a significant number of fatalities. Heart disease, stroke and other CVDs are believed to be responsible for 17.9 million deaths worldwide. Particularly, 33% of these deaths happen to those under 70 highlighting the seriousness of CVD’s influence on world health. The European Cardiology Society reports that there are 26 million heart disease sufferers with 3.6 million new cases every year. The condition is more prevalent among individuals who smoke, have hypertension, are obese, suffer from diabetes and engage in insufficient physical activity. Early detection is essential in order to effectively treat and avoid negative consequences like heart failure and stroke (Hassan et al., 2024).

When it comes to complex CVD situations, traditional diagnostic methods may not be as accurate. Machine Learning (ML) has emerged as a powerful method for predicting cardiac disease. The ability of ML technique to increase prediction accuracy has been validated by many studies. ML approaches, including hybrid models, real time data and advanced preprocessing techniques have been found in recent study and have demonstrated promising results in terms of increasing accuracy and performance (Srinivasan et al., 2023).

For the classification of various cardiac abnormalities, an ensemble model that combines Deep Learning (DL) and ML algorithms performs better than baseline methods (Hasan et al., 2025). One of the main areas of current study is the combination of feature selection, DL and ML techniques with optimization techniques to further enhance performance. Through these enhancements, models will be improved, their clinical applicability will be advanced and their efficiency in real-world situations will be confirmed.

The proposed work combines several new techniques to improve accuracy and robustness, introducing a hybrid approach for heart disease prediction. The RST-QRDM approach is utilized for optimal feature selection, ensuring a smaller and more effective feature set. The selected features are transformed into an RNS representation that successfully captures the vagueness and uncertainty seen in the data. To minimize redundancy and extract features, a OptiDANet model is used. To further optimize feature extraction, Optuna an advanced hyperparameter tuning framework is employed to fine tune the OptiDANet model. The extracted features are classified using a RF model ensuring robust and interpretable decision making. This integration of RST, RNS, OptiDANet and RF presents a new and comprehensive approach to heart disease prediction addressing key challenges in uncertainty handling, feature selection and classification thereby outperforming existing models in accuracy and reliability. The key contributions of this research are as follows,

- RST-QRDM approach identified the most significant features thereby reducing dimensionality.
- Selected features are transformed into RNS representation capturing uncertainty.
- The OptiDANet model with CAM and SAM enhances the feature extraction and performance.
- Extracted features are classified using RF ensuring high accuracy and interpretability with the proposed RNS-OptiDANet model outperforming existing models.

The proposed RNS-OptiDANet framework provides a strong, uncertainty aware and optimized predictive result for clinical heart risk assessment. The paper is organized as follows. Section 2 reviews related literature, section 3 explains the proposed approach, section 4 presents experimental results and discussions and section 5 conclude the study with recommendations for future research.

## 2 Review of related studies

ML has significantly enhanced the prediction of heart disease by obtaining higher accuracy and early diagnosis using efficient classifiers and hybrid feature selection. A summary of existing models for predicting cardiac disease and the use of integrated approaches in various fields are given in this section.

By highlighting the significance of accurate feature selection and strong classification models, new developments in ML have significantly enhanced the prediction of heart disease. Research has confirmed that even comparatively basic supervised classifiers such RF, DT and KNN can attain superior sensitivity, specificity and accuracy in this field (Ali et al., 2021). More advanced methods have combined deep convolutional neural networks (DCNNs), recursive feature elimination and genetic algorithms (GA) with metaheuristic techniques like Elephant Herd Optimization to improve predictive performance and solve data imbalance problems (Al-Alshaikh et al., 2024). Besides model based developments, studies have also observed into the genetic and biochemical links between heart disease and other conditions using computational biology methods to find important gene interactions and possible treatment targets (Xu et al., 2024).

Building on these developments RST has been applied recently as a practical feature selection method particularly when dealing with high dimensional data that incorporates redundant features. It has been shown through the application of RST to diverse datasets that redundant features can be removed without causing significant loss of information improving classification performance across a variety of ML models. This proves that RST based feature selection not only improves model performance but also lowers computational complexity (Ashika and Grace, 2025).

The combination of ML and XAI techniques has led to notable improvements in the prediction of heart disease. The prediction accuracy of hybrid feature selection and classifiers like XGBoost (XGB) and RF has reached 97.57% while SHAP analysis has found important risk factors including cholesterol and age (El-Sofany et al., 2024). In the same way, ML techniques such as Gradient Boosting and CatBoost have proven to be highly accurate in forecasting early-stage CVD enabling quick clinical interventions (Baghdadi et al., 2023).

For the purpose of predicting heart disease, a sparse guided attentive architecture was created that uses multi-stage feature selection, data balance and outlier identification. The model achieves good accuracy with less features when tested on datasets such as UCI, Framingham and Faisalabad (Altantawy and Kishk, 2024). The GAPSO-RF method improves feature selection and attains accuracies of 95.6 and 91.4% by combining particle swarm optimization (PSO) and GA with RF. Future improvements include CNN for feature selection and multi-objective GAs, despite their computational cost (El-Shafiey et al., 2022).

By tackling uncertainty in huge healthcare datasets, a hybrid model that combines Cuckoo Search and RST approaches (CSRS) has been developed to improve the diagnosis of heart disease. While RST generates decision rules with an accuracy of 93.7%, CS optimizes feature selection. Future research in fuzzy and intuitionistic RST is recommended for wider applicability as the model also produces 32.91% less rules increasing decision-making efficiency (Acharjya, 2020). A group incremental feature selection technique that combines RST with GAs uses previously calculated reducts to identify important features in dynamic datasets while lowering computational cost. This method works well on benchmarks reducing computation time while preserving excellent classification accuracy which makes it appropriate for dynamic applications such as large data and bioinformatics (Das et al., 2018).

For high dimensional datasets, a hybrid feature selection method that combines RST and PSO efficiently strikes a compromise between feature dependencies and optimal search enhancing ML accuracy and efficiency (Wang et al., 2007).

A stability theorem is incorporated into RST-based frameworks in an attempt to increase algorithm speed, accuracy and noise resistance addressing scalability for big datasets through parallel processing (Xia et al., 2022). By transforming datasets into forest structures, the feature forest approach optimizes feature selection while greatly increasing computational efficiency and storage needs particularly for datasets with a large number of features (Wang and Ma, 2009). Important variables for heart disease prediction are also identified by a computational intelligence based feature selection technique based on RST increasing the model’s effectiveness and accuracy (Prabha et al., 2021).

RST and neutrosophic set theory (NST) offer powerful tools for controlling uncertainty in data processing. In order to improve computational effectiveness and address insufficient information, recent research has focused on improving data preparation through effective attribute reduction. With hybrid models that combine ML and neutrosophic representations, this method has demonstrated a notable impact in fields such as medical diagnostics (Zhang et al., 2020; Ashika et al., 2024). The NST-ML technique when applied to the Wisconsin Diagnostic Breast Cancer (WDBC) dataset produced a 99.12% accuracy rate using N-AdaBoost indicating its potential for accurate diagnostics (Zainal et al., 2023).

In order to increase accuracy while reducing processing complexity a study suggested a lightweight 1D CNN model for environmental sound classification that makes use of CAM and Time Attention Module (TAM). TAM concentrates on discriminative temporal aspects whereas CAM captures frequency-related dependencies. By combining these modules a Time-Channel Attention Module (TCAM) is created that can process raw audio waveforms directly and recalibrate feature maps without the requirement for feature pre-extraction. The model performs better than existing 1D CNNs and produces superior results when compared to 2D CNNs making it perfect for real time applications in embedded systems according to evaluations on the UrbanSound8k and ESC-10 datasets (Xu et al., 2024).

SAM is used to find pertinent regions in huge Whole-Slide Image (WSI) data using a Dual Attention Model for Histology WSI classification. SAM highlights the most important regions through an attention map improving computational efficiency. A hard attention model extracts multi-resolution glimpses from these regions further enhancing the classification process while reducing computation by over 75% (Raza et al., 2024).

Recent advancements in hyperparameter optimization have led to the development of several models that leverage the Optuna framework for various applications. The Optuna driven Deep Fuzzy Neural Network (Optuna-DFNN) model integrates DL, Optuna optimization and FNN techniques to enhance performance metric prediction. It addresses challenges like parameter tuning, self-learning capabilities and generalization achieving high accuracy in predicting key metrics crucial for blast furnace production (Li et al., 2024). The Hybridized Optuna Optimization XGB (hyOPTXg) expert system employs Optuna for XGB hyperparameter optimization to predict CVD reducing overfitting and enhancing model reliability through dynamic parameter exploration and pruning strategies (Srinivas and Katarya, 2022).

Optuna’s CNN Long Short-Term Memory (CNN-LSTM) model outperformed more conventional optimization techniques like PSO in energy consumption predictions (Ekundayo, 2020). In order to improve healthcare systems for individualized risk assessments, the HyOPTRF and HyOPTXGB models combine Optuna with RF and XGB for early heart disease prediction attaining high accuracy (Dhanka and Maini, 2024).

While these methods report strong predictive performance through optimization, ensemble learning and explainability, they largely overlook explicit uncertainty modeling which motivates the proposed framework that integrates RST and NST based uncertainty handling with optimized ML for more reliable and interpretable heart disease prediction. Table 1 provides an overview of related studies on heart disease prediction highlighting the techniques, methodologies, results and key inferences from each work.

**Table 1:** Summary of related work on heart disease prediction.

References	Technique	Methodology—results	Inference
El-Sofany et al. (2024)	Hybrid Feature Selection and Classifiers	XGB and RF achieved 97.57% accuracy; Important risk factors like age and cholesterol were found by SHAP analysis.	Facilitates early detection and personalized risk assessment.
Baghdadi et al. (2023)	Gradient Boosting and CatBoost	Achieved high accuracy in early-stage CVD prediction supporting timely clinical interventions.	ML methods enhance predictive accuracy for early CVD diagnosis.
Altantawy and Kishk (2024)	Sparse-Guided Attentive Architecture	Uses outlier detection, data balancing and multi-stage feature selection. Evaluated on UCI, Framingham and Faisalabad datasets with high accuracy.	Achieves high accuracy using fewer features optimizing computational efficiency.
Prabha et al. (2021)	Computational Intelligence based Feature Selection	Identifies crucial variables for heart disease prediction, improving accuracy and efficiency.	Enhances model performance in predictive tasks.
Srinivas and Katarya (2022)	hyOPTXg	Employs Optuna for XGB hyperparameter tuning improving CVD prediction reliability.	Reduces overfitting and enhances model generalization.
Dhanka and Maini (2024)	HyOPTRF & HyOPTXGB	Combines Optuna with RF and XGB for heart disease prediction achieving high accuracy.	Enhances personalized risk assessments in healthcare.

A thorough review of the existing literature reveals a gap in research as no prior studies have specifically addressed the methodology proposed for heart disease prediction in this work. While numerous studies have explored traditional ML and DL approaches for heart disease diagnosis none have combined RST and RNS for feature selection and representation along with OptiDANet for feature extraction. While transformer-based architectures and deep attention models have recently gained popularity in medical prediction most are optimized for large scale imaging or sequential data. Because of the limited direct applicability to structured tabular datasets, the proposed RNS-OptiDANet model is instead compared to powerful ensemble attention-based methods and uncertainty-aware approaches. This difference highlights the uniqueness and significance of the proposed RNS-OptiDANet approach which makes use of these advanced mathematical methods to increase prediction accuracy and interpretability.

## 3 Methodology and techniques

This section covers dataset preprocessing, RST-QRDM approach for feature selection, RNS feature generation, OptiDANet model for feature extraction, RF classification, and performance evaluation.

### 3.1 Proposed RNS-OptiDANet approach

**Figure 1:**
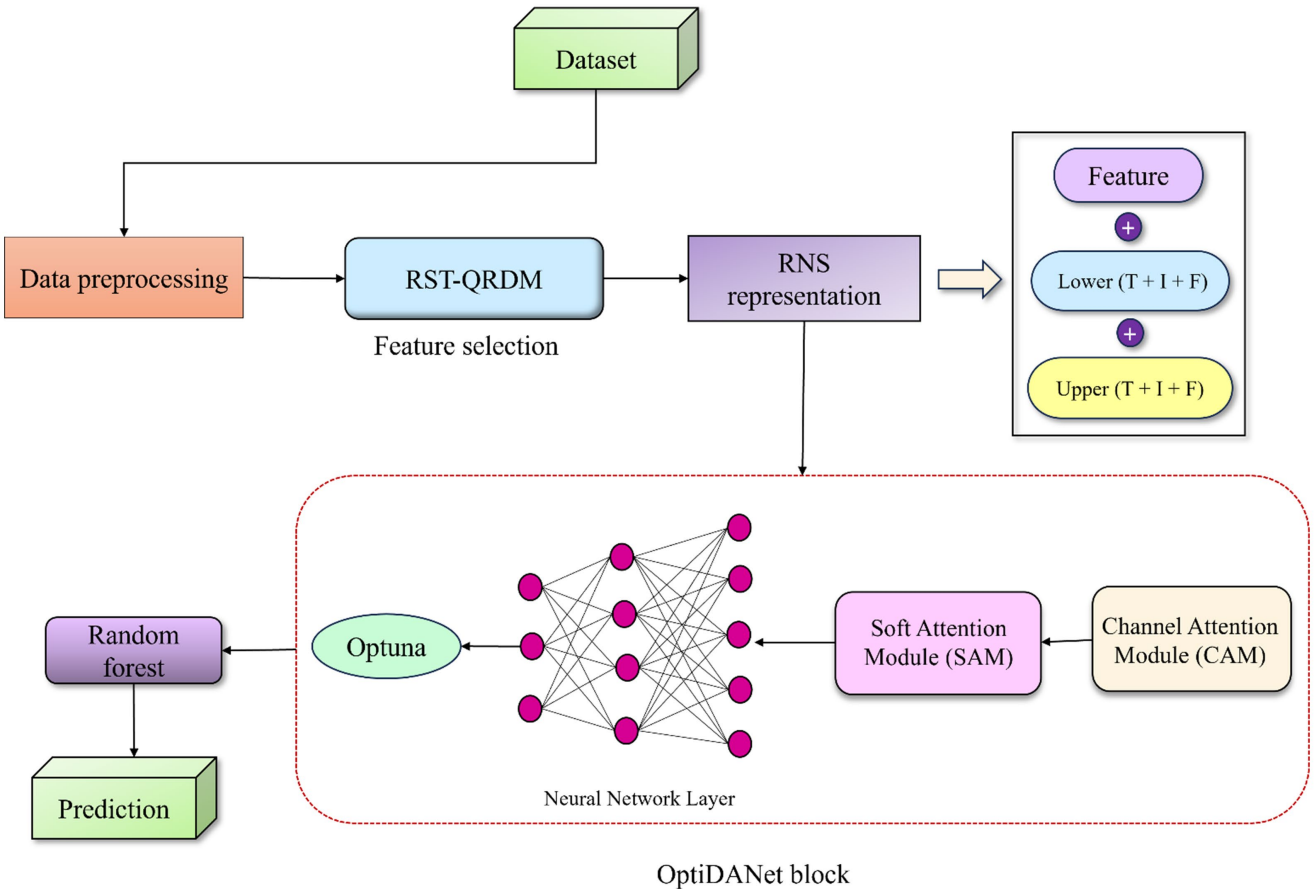
Overview of the proposed RNS-OptiDANet model.

The proposed RNS-OptiDANet approach was used to evaluate the dataset on heart disease (Srinivas and Katarya, 2022) in order to provide precise predictions. For uniform feature scaling, the dataset was scaled during the preparation stages. This scaled dataset is then subjected to feature selection using RST-QRDM approach resulting in a minimal set of significant features. The selected features are transformed into the RNS representation by computing lower and upper approximations for each feature capturing uncertainty and vagueness. These approximations are combined to generate the RNS feature set which serves as input for the proposed OptiDANet model. The OptiDANet model is employed for feature extraction with its hyperparameters optimized using an advanced tuning framework, Optuna to improve training efficiency leading to a refined model with enhanced feature extraction. The performance of the RF model is evaluated using accuracy ( $A_{\mathrm{cc}}$ ), precision ( $P_{\mathrm{rn}}$ ), recall ( $R_{\mathrm{cl}}$ ) and F1-score ( $F_{1s}$ ) based on the features extracted by the OptiDANet model. Figure 1 depicts the architecture of the proposed RNS-OptiDANet framework.

### 3.2 Data preprocessing

The dataset was first split into training and testing subsets to ensure unbiased evaluation and all preprocessing was performed after the split to prevent data leakage. Categorical variables were encoded using label encoding for binary features and one-hot encoding for multiclass features. Due to heterogeneous feature scales, z-score standardization was applied so that each feature has zero mean and unit variance ensuring balanced contribution during RNS computation. The standardization is expressed as in Equation 1.

$$X_{\mathrm{std}}=\frac{x-\mu_{\text{train}}}{\sigma_{\text{train}}}$$ (1)

Where $\;\mu_{\text{train}}$ and $\sigma_{\text{train}}$ are computed using the training data. The scaler was fitted only on the training set and applied to the test set with the same training derived statistics reused within each cross-validation fold. All subsequent steps including RNS transformation and OptiDANet feature extraction used these training fitted parameters.

### 3.3 Enhanced QuickReduct algorithm for feature selection using discernibility matrix

Proposed by Stańczyk and Zielosko (2020) and Walczak and Massart (1999), RST is a mathematical approach for managing imprecise and inconsistent data through approximation based granular structures. RST supports feature selection by identifying minimal subsets of attributes known as reducts that preserve the classification capability of the dataset. Unlike the conventional QuickReduct algorithm which relies solely on dependency measures to select attributes contributing to classification, the proposed RST-QRDM approach integrates a discernibility matrix to guide feature selection. The proposed RNS-OptiDANet framework was used to evaluate the dataset on heart disease (Srinivas and Katarya, 2022) in order to provide precise predictions. To guarantee uniform feature scaling, the dataset was scaled during the preparation stages.

By reducing the search space for reducts and promoting the selection of a minimum yet discriminative feature set, the discernibility matrix increases computational efficiency. The QRDM approach not only speeds up the reduct computation process but also improves model performance by offering a more consistent and clear classification foundation.

The RST-QRDM algorithm selects features based on their ability to maximally reduce the discernibility matrix ensuring that all object pairs with different class labels are distinguishable. The selection process continues until either all distinctions are resolved or a predefined maximum number of features is reached. Algorithm for the RST-QRDM method is presented in Algorithm 1.

ALGORITHM 1 RST-QRDM approach. 
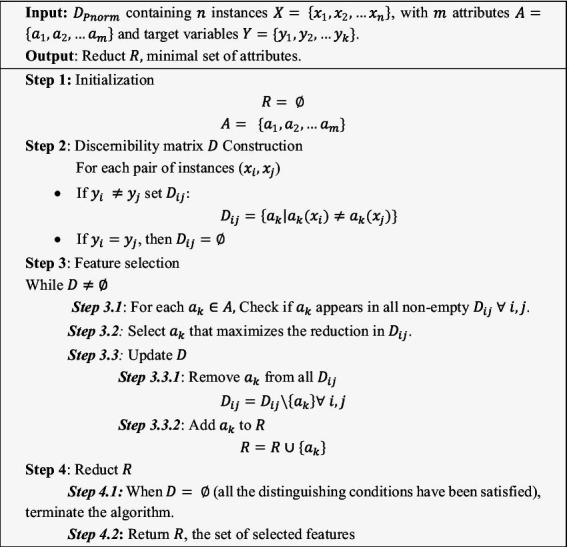

### 3.4 Generation of rough neutrosophic feature

Each selected feature is transformed into RNS representation by computing its lower and upper approximations of Truth (T), Indeterminacy (I) and Falsity (F). The RNS transformation assumes the presence of uncertainty, indeterminacy and vagueness in the input data. RNS enable feature representation by decomposing features into six distinct components: lower membership $\mu_{T_{*}}$ _(NS˛)_ (*x*), lower indeterminacy $\nu_{T_{*}}$ _(NS˛)_ (*x*), lower non-membership $\omega_{T_{*}}$ _(NS˛)_ (*x*), upper membership $\mu_{T^{*}}$ _(NS˛)_ (*x*), upper indeterminacy $\nu_{T^{*}}$ _(NS˛)_ (*x*) and upper non-membership $\omega_{T^{*}}$ _(NS˛)_ (*x*). This method effectively represents the degrees of association, uncertainty and disassociation within the heart disease data providing a robust framework for managing imprecision and ambiguity in ML models (Broumi et al., 2014).

The RNS representation of the selected heart disease features using RST-QRDM approach captures the inconsistency, incompleteness and uncertainty contained in the data. RNS calculates lower and upper approximations, including T, I and F measurements for every feature. The degrees of certainty that an item belongs to the feature set or does not are represented by the lower approximations ( $\mu_{T_{*}}$ _(NS˛)_ (*x*) ν_T*__(NS˛)_ (*x*) ϖ_T*__(NS˛)_ (*x*)). The upper approximations ( $\mu_{T^{*}}$ _(NS˛)_ (*x*) ν_T*__(NS˛)_ (*x*) ϖ_T*__(NS˛)_ (*x*)) extend these measures by considering objects that possibly belong to the feature set.

This transformation expands the original feature space multiplying the number of features by six. Although this increases dimensionality, it enhances the input representation by incorporating boundary and uncertainty characteristics of the data thereby improving the model’s capacity to learn complex patterns. The inclusion of these six RNS components allows the model to handle imprecision, vagueness and boundary regions in the data which is critical in medical prediction tasks such as heart disease classification where data ambiguity is common. The Algorithm for the RNS feature generation is presented in Algorithm 2.

ALGORITHM 2 Feature generation using RNS. 
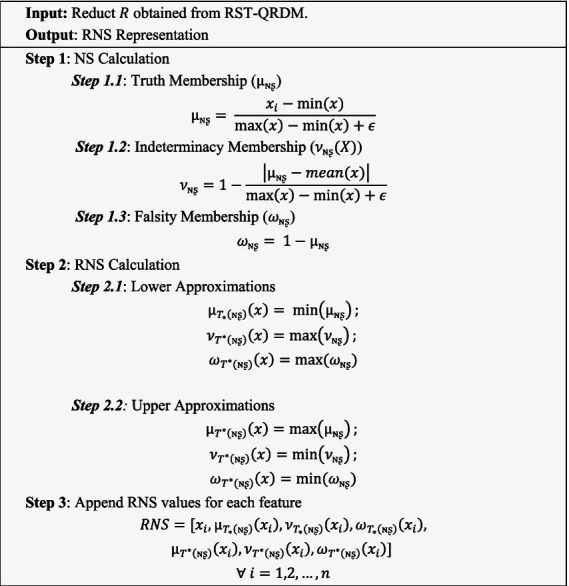

### 3.5 Feature extraction using optimized dual attention neural network model

The proposed OptiDANet model processes the RNS feature set incorporating lower and upper approximations. The OptiDANet model integrates both CAM and SAM to leverage their complementary advantages. CAM recalibrates channel wise feature importance to highlight critical factors, while SAM captures dynamic inter-feature relationships via Query (Q), Key (K) and Value (V) attention. Together they improve the model’s feature learning process leading to enhanced robustness and predictive efficiency.

The OptiDANet model integrates fully connected layers with CAM and SAM to facilitate effective feature extraction. After training feature embeddings are extracted and used to train a RF Classifier which is then evaluated on test data. Optuna performs hyperparameter tuning across 50 trials after which the optimized model is reloaded, features are re-extracted and the RF Classifier is retrained for final evaluation.

**Figure 2:**
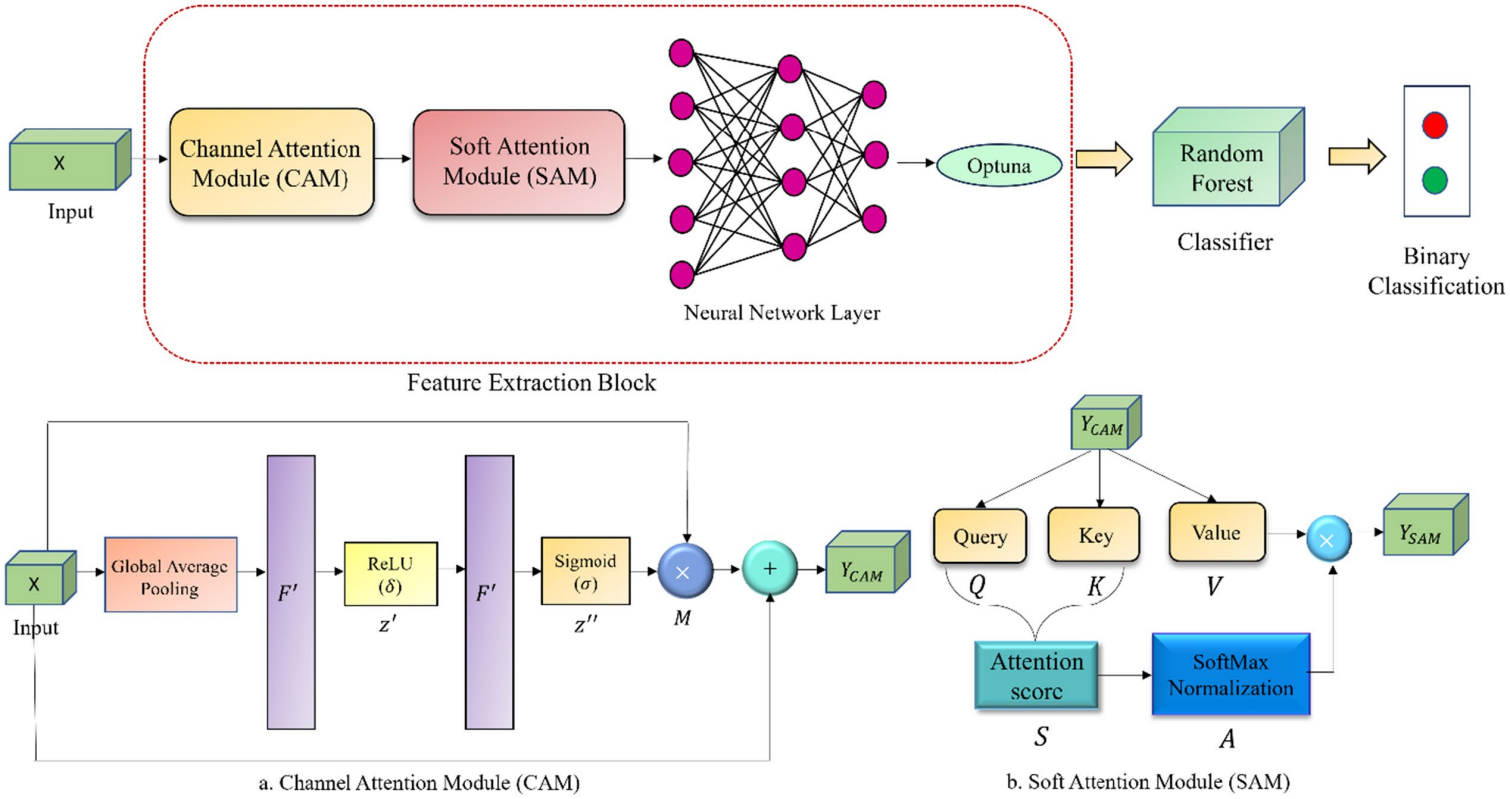
Workflow of the OptiDANet model showing **(a)** the CAM layer and **(b)** the SAM layer.

The following subsections details the foundation of the OptiDANet model. The workflow of the proposed OptiDANet model highlighting the structures of the CAM and SAM is illustrated in Figure 2.

#### 3.5.1 Channel attention model (CAM)

CAM assigns adaptive importance weights to each feature channel reducing the influence of less informative features while emphasizing the most relevant ones. The input feature map is first processed using adaptive average pooling along the sequence length to obtain channel wise summary statistics. These statistics are refined through a gating mechanism consisting of two fully connected layers implemented using 1 × 1 convolutions. The first layer followed by a ReLU activation which introduces non-linearity while the second layer applies a sigmoid activation to generate normalized channel attention weights. The resulting weights are used to recalibrate the input feature map through element wise multiplication followed by a residual connection to preserve the original feature information. By improving the discriminative channel representations, CAM increases the overall performance of the model (Xu et al., 2024).

#### 3.5.2 Soft attention model (SAM)

SAM assigns relevance weights across the input sequence dynamically thereby improving the model’s capacity to understand contextual dependencies. Learnable weight matrices are used to linearly change the recalibrated feature map into Q, K and V representations. To maintain numerical stability, attention scores are scaled by the square root of the feature dimension and calculated by comparing Q and K. The SoftMax function is used to scale these scores and create attention weights that show how important several sequence components are in relation to one another. By adding these weights to V, the final output is formed which enables the model to efficiently aggregate relevant contextual information. The model’s representational capacity is enhanced by this soft attention mechanism which allows it to suppress less important features while concentrating on salient ones (Raza et al., 2024).

#### 3.5.3 Optuna

The RNS-OptiDANet model was optimized using Optuna, an open-source hyperparameter optimization framework. By using powerful search and pruning techniques, this framework automates the process of identifying the best parameters for the RNS-OptiDANet model facilitating faster and less expensive optimization. Because of its adaptability, it can be used for both large-scale distributed computing and small-scale studies (Akiba et al., 2019).

Optuna was added to the RNS-OptiDANet model’s training process to enhance the neural network training process. The goal function improved several neural network and classifier hyperparameters, such as learning rate, batch size, regularization strength, network width and important RF parameters by training the model over a predetermined number of epochs and monitoring accuracy and loss. Over the course of 50 trials, the hyperparameter tuning procedure was carried out. After that, the RF classifier was retrained for the final assessment, features were reextracted and the optimal model was reloaded. This process stops information from leaking during assessment by making sure hyperparameter optimization does not reuse or adjust to the test set. The Optuna search space and the final hyperparameter values chosen for configuring the shared OptiDANet feature extractor and related models are summarized in Table 2. To guarantee consistency in evaluation across classifiers and reproducibility these parameters were established for every experiment.

**Table 2:** Hyperparameters optimized using Optuna.

Component	Hyperparameter	Search range
Neural network	Learning rate	1e−5 – 1e−1 (log scale)
	Batch size	16–128
	Weight decay	1e−6 – 1e−2
	Hidden layer expansion	{2×, 4×}
Random forest	Number of trees	50–300
	Maximum depth	5–30
	Minimum samples split	2–10

The proposed RNS-OptiDANet architecture was manually designed based on insights from previous attention-based models and the specific structure of RNS features. The design aimed to balance complexity and interpretability making use of CAM and SAM layers to effectively capture both channel-wise and contextual dependencies. Automated architectural search was not employed to maintain control over model interpretability and computational cost.

#### 3.5.4 Random forest

In this study OptiDANet is employed as a deep feature extractor while classification is performed using a RF classifier. This hybrid design combines uncertainty aware deep representations learned by OptiDANet with the robustness, generalization capability and interpretability of ensemble learning rather than relying on a purely end-to-end softmax based deep classifier. The RF model an ensemble learning technique that aggregates the results of several DT models to increase predictive accuracy and decrease overfitting. A bootstrap sample of the dataset is used to train each tree increasing diversity and decreasing inter-tree correlation. At each node a randomly selected subset of features is assessed allowing the trees to capture unique patterns within the data.

The RF model uses averaging for regression problems and majority voting for classification tasks to aggregate predictions. This method efficiently manages complicated datasets and improves model stability. The RF model, known for its high predictive accuracy and ability to mitigate overfitting works with the enhanced OptiDANet architecture resulting in a powerful and strong predictive framework (Chaudhary et al., 2016; Breiman, 2001).

### 3.6 Evaluation metrics

Performance metrics such as $A_{\mathrm{cc}}$ , $P_{\mathrm{rn}}$ , $R_{\mathrm{cl}}$ and $F_{1s}$ are used to assess performance. The percentage of subjects that are correctly classified is denoted by $A_{\mathrm{cc}}$ . The percentage of subjects correctly classified as positive out of all positive subjects is known as $P_{\mathrm{rn}}$ . The model’s capacity to identify positive samples is assessed by $R_{\mathrm{cl}}$ . The $F_{1s}$ is a harmonic mean of recall and precision. Table 3 provides specifics on the evaluation metrics.

**Table 3:** Evaluation metrics formulae.

Evaluation metrics	$A_{\mathrm{cc}}$	$P_{\mathrm{rn}}$	$R_{\mathrm{cl}}$	$F_{1s}$
Formulae	$\frac{\left(\mathrm{TN}+\mathrm{TP}\right)}{\left(\mathrm{TN}+\mathrm{FN}+\mathrm{TP}+\mathrm{FP}\right)}$	$\frac{\left(\mathrm{TP}\right)}{\left(\mathrm{TP}+\mathrm{FP}\right)}$	$\frac{\left(\mathrm{TP}\right)}{\left(\mathrm{TP}+\mathrm{FN}\right)}$	$2\times \frac{R_{\mathrm{cl}}\times P_{\mathrm{rn}}}{R_{\mathrm{cl}}+P_{\mathrm{rn}}}$

Where TP = True Positives, TN = True Negatives, FP = False Positives and FN = False Negatives.

## 4 Results and discussions

This section outlines the experimental results as well as the dataset characteristics, feature selection methods and preprocessing techniques. It reports the performance of the enhanced RNS-OptiDANet model and details the ML training process. A thorough ablation study is conducted to analyze the contribution of individual components including baseline ML models, RST and RNS based feature representations and their integration with OptiDANet. The study further examines the impact of feature cardinality and identifies the optimal configuration for the RNS-OptiDANet framework using a TOPSIS based Multi-Criteria Decision-Making (MCDM) approach. Comparative evaluations against existing heart disease prediction methods and validation across multiple open-source datasets are provided to assess robustness and generalizability. SHAP and LIME based interpretability analyses are presented followed by a discussion of the results and study limitations.

### 4.1 Experimental setup

The proposed RNS-OptiDANet model was implemented in Google Colab, a cloud-based Jupyter notebook environment using PyTorch. Training was conducted on a system equipped with a $12^{\mathrm{th}}$ Gen Intel Core i3-1215U processor (1.20 GHz) and 8 GB RAM. Parameter updates were carried out with the Adam optimizer initialized with a learning rate of 0.001. To ensure effective heart disease prediction the batch size and other hyperparameters were optimized using Optuna. The OptiDANet model was trained for 50 epochs with a batch size of 32.

For performance evaluation the dataset was split into training (70%) and testing (30%) subsets. Cross validation was not employed instead model generalizability was assessed through repeated Optuna trials.

For all three benchmark datasets [Obesity, Breast Cancer and chronic kidney disease (CKD)], the same model configuration and hyperparameter settings optimized using Optuna on the heart disease dataset were retained. The goal of this method was to assess the model’s robustness and generalizability across several healthcare areas.

### 4.2 Dataset description

The heart disease dataset used in this study is publicly available in Kaggle (Li et al., 2024). The original dataset comprises records collected from four clinical sources namely Cleveland, Hungary, Switzerland and Long Beach V and contains 76 attributes including the target variable. Consistent with standard practice in the literature and prior published studies only a subset of 14 clinically relevant attributes is considered for experimental evaluation. This subset consists of 13 predictive features and one target variable indicating the presence (1) or absence (0) of heart disease. No manual feature elimination was performed at this stage.

Although earlier studies and individual UCI subsets such as the Cleveland dataset contain 303 samples the dataset employed in this work corresponds to the consolidated version comprising 1,025 samples obtained by merging records from multiple clinical sources. All experiments were conducted using the full dataset and no sample level reduction was applied.

**Figure 3:**
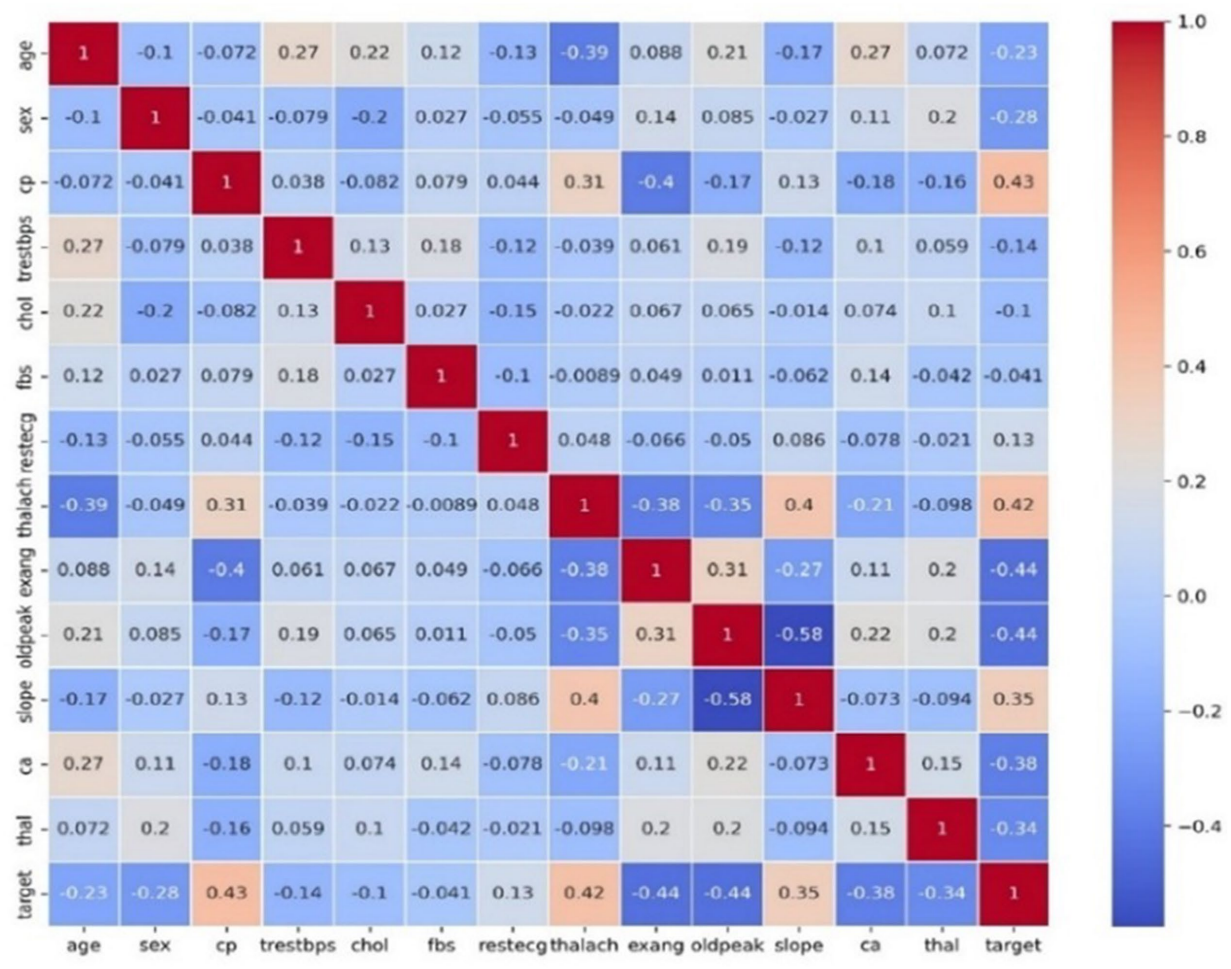
A heatmap of the dataset on heart disease.

A heat map showing the correlation matrix of the characteristic of the dataset is shown in Figure 3. A visual representation of the direction and strength of the correlations between feature pairs is provided by the color intensity. This heat map is a useful tool for understanding intricate interactions between features and provides information for further stages of modeling and data preprocessing.

### 4.3 Data preprocessing and feature selection

The dataset underwent preprocessing revealing no missing values or categorical data requiring encoding. It was divided into 70:30 ratio for training and testing. The results were scaled between 0 and 1 which is the required range for RNS using min-max normalization.

Feature dimensionality reduction is performed algorithmically using RST and not through manual dataset pruning, ensuring objective and reproducible feature selection. Specifically, the RST-QRDM approach is applied exclusively on the training data to identify the most informative attributes from the initial 14 feature set. Five important characteristics including age, gender, fasting blood sugar (fbs), resting blood pressure (trestbps) and chest pain type (cp) were chosen as a result of the RST-QRDM approach. By efficiently reducing dimensionality these features enable better classification performance by capturing the most discriminative patterns in the data.

RNS was used to expand each feature into six attributes that represent lower and higher approximations across T, I and F after the RST feature selection process. All of the features that were chosen undergo this modification in a similar manner. By successfully resolving ambiguity and vagueness in the data, the resulting RNS based features enhance the representation of the dataset and increase the robustness and accuracy of the model. They also successfully capture the lower and upper approximations for T, I and F.

### 4.4 Result of the enhanced RNS-OptiDANet model

The OptiDANet model which combines CAM and SAM to refine input features by lowering noise and highlighting essential features addressing uncertainty and ambiguity for better classification uses the RNS converted data enhanced with T, I and F approximations as input. The RNS-OptiDANet model is trained on a training dataset that has Optuna optimized hyperparameters like batch size and learning rate. Only the training data was used for hyperparameter optimization using Optuna and the chosen hyperparameters were fixed before cross validation and applied consistently across all folds.

After training, the RNS-OptiDANet model produces useful data representations that capture key features. These features are then used as input by the RF classifier enabling a comprehensive evaluation of their efficacy. The enhanced OptiDANet model provides an effective hybrid technique for reliable and accurate classification by fusing the characteristics of classical ML models with the feature extraction capabilities of DL models.

**Figure 4:**
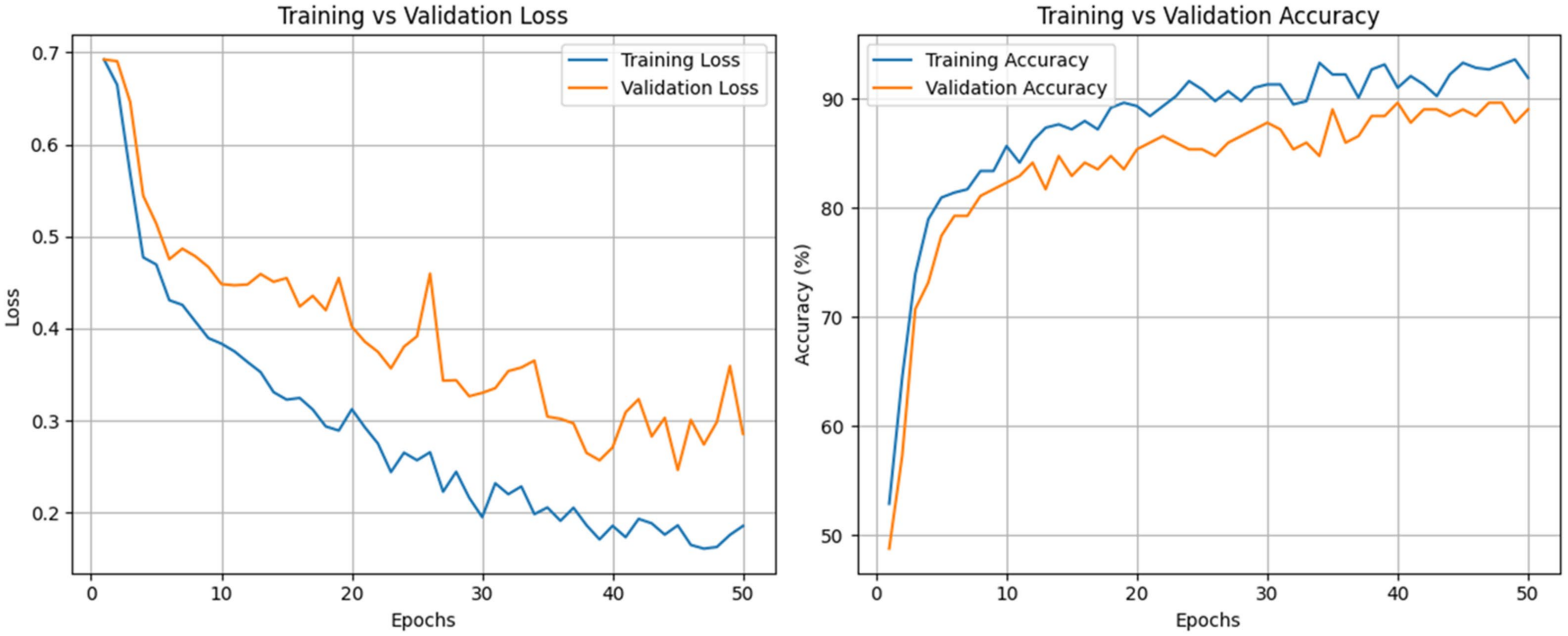
Training vs. validation loss and accuracy of the RNS-OptiDANet model.

Figure 4 illustrates the training and validation learning curves of the proposed RNS-OptiDANet model. Both loss curves decrease consistently with no late-stage increase in validation loss while training and validation accuracy improve in parallel with only a small and stable generalization gap of approximately 3–5%. The absence of divergence between the curves indicates that the model learns representative patterns rather than memorizing the training data demonstrating controlled model complexity and good generalization behavior.

The features generated from the RNS-OptiDANet model with hyperparameters optimized using Optuna are taken as input for training the RF model.

RF known for its reliability and versatility leverages ensemble learning by aggregating predictions from multiple DTs, effectively capturing complex data patterns while reducing overfitting. Metrics including $A_{\mathrm{cc}}$ , $P_{\mathrm{rn}}$ , $R_{\mathrm{cl}}$ and $F_{1s}$ are used to evaluate the RF model’s performance in order to determine how well it predicts outcomes and how robust it is to test data. Table 4 shows the experimental results of the proposed RNS-OptiDANet model.

**Table 4:** Evaluation results of the proposed RNS-OptiDANet model.

Evaluation metrics	Performance of RF classifier
$A_{\mathrm{cc}}$	99.51%
$P_{\mathrm{rn}}$	99.52%
$R_{\mathrm{cl}}$	99.51%
$F_{1s}$	0.9951

**Figure 5:**
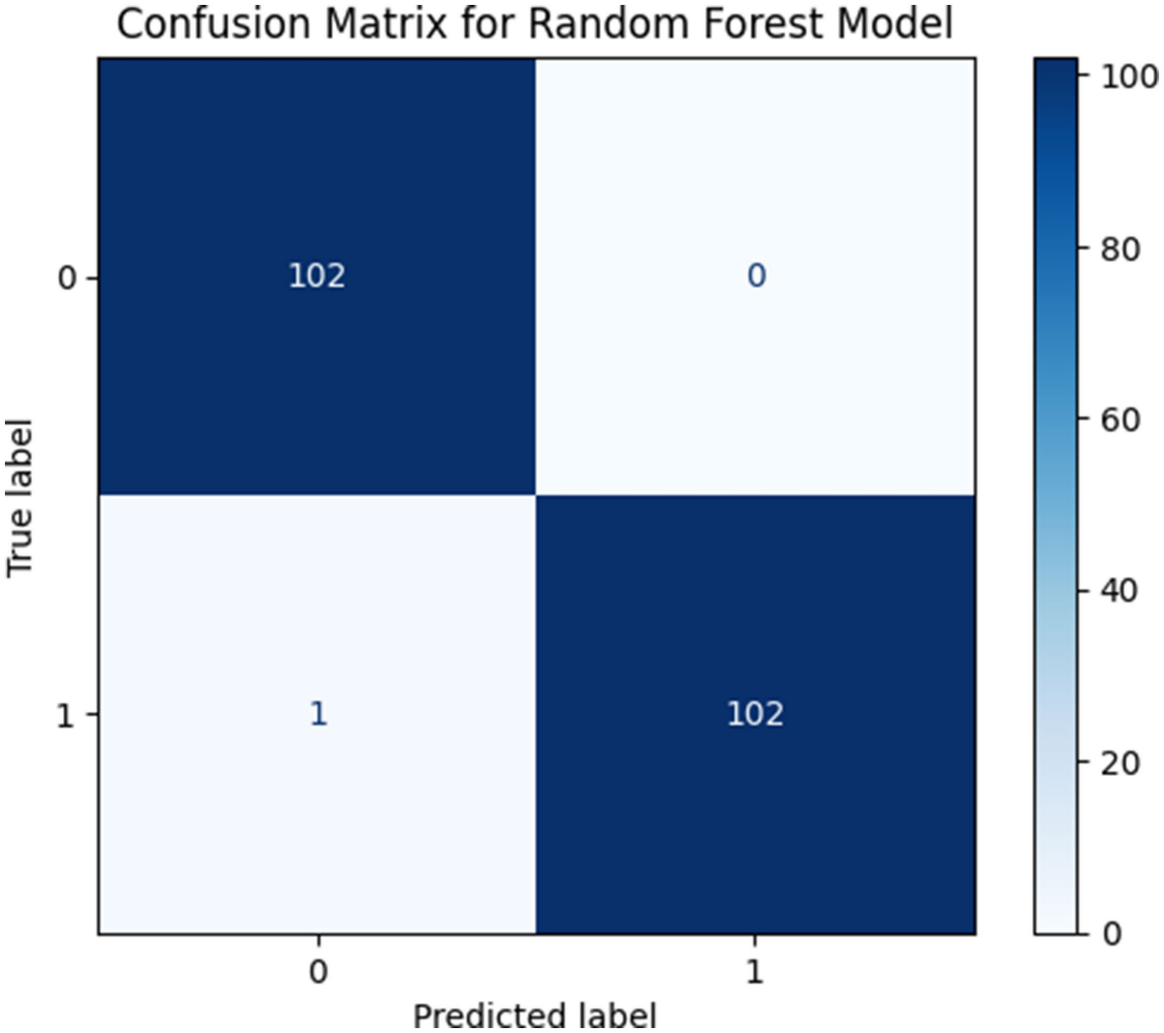
Confusion matrix of the proposed RNS-OptiDANet model.

The proposed RNS-OptiDANet model demonstrated outstanding performance achieving an accuracy of 99.51% which highlights its reliability and precision for the classification of heart disease. The confusion matrix of the proposed RNS-OptiDANet model for heart disease dataset is shown in Figure 5.

To further assess generalization and mitigate overfitting concerns 5-fold stratified cross validation was conducted on the heart disease dataset. The complete RNS-OptiDANet pipeline was trained independently in each fold using fixed Optuna optimized hyperparameters with feature scaling and model training confined to the respective training folds. The proposed model achieved a mean accuracy of 98.15% ± 0.57 indicating stable performance across data splits and confirming that the results are not dependent on a specific partition. Table 5 summarizes the 5-fold cross-validation results.

**Table 5:** 5-fold cross-validation results of the proposed RNS-OptiDANet model.

Metric	Mean ± Std
$A_{\mathrm{cc}}$	98.15 ± 0.57
$P_{\mathrm{rn}}$	0.9817 ± 0.0054
$R_{\mathrm{cl}}$	0.9816 ± 0.0058
$F_{1s}$	0.9815 ± 0.0057

A computational cost analysis was performed to compare the baseline RF model with the proposed RNS-OptiDANet framework. The baseline RF achieved 98.54% accuracy with a training time of 0.23 s and inference time of 0.0481 ms/sample. In contrast the proposed model achieved higher accuracy of 99.51% at the cost of increased training time of 1.28 s due to deep feature extraction while maintaining low inference latency of 0.1889 ms/sample. Table 6 shows the comparison of accuracy and computational cost.

**Table 6:** Accuracy and computational cost comparison.

Model	$A_{\mathrm{cc}}$ (%)	Training time (s)	Inference time (ms/sample)
Baseline RF	98.54	0.23	0.0481
**RNS-OptiDANet**	**99.51**	**1.28**	**0.1889**

Bold values indicate the best performance within the table.

### 4.5 Ablation study

To examine the role of individual components, an ablation analysis was carried out on the proposed method. The research compared baseline ML models and assessed the performance of RST selected features with ML, RST combined with RNS and ML and RST with RNS integrated into the OptiDANet mechanisms for feature extraction followed by ML. The models analyzed include Logistic Regression (LR), KNN, SVM, Naive Bayes (NB), DT, RF, XGB, AdaBoost (ADB), LightGBM (LGBM) and Extra Trees (ET). This study confirms that each element is required to ensure an effective and efficient final model architecture for the task. The ablation study focuses on isolating the impact of OptiDANet based feature extraction by comparing the proposed hybrid framework against a direct RF classifier trained on RNS features without deep representation learning. The ablation results further support the generalization capability of the proposed framework by demonstrating consistent performance degradation when individual components are removed.

#### 4.5.1 Component-wise ablation analysis

This subsection systematically evaluates the contribution of each component within the proposed RNS-OptiDANet framework. It begins with baseline ML performance followed by analysis of RST based feature selection and RNS representation. The impact of the attention modules such as CAM and SAM is then examined. Model selection is performed using the MCDM-TOPSIS approach followed by a summary comparison and discussion of all framework variants.

##### 4.5.1.1 Baseline ML model performance evaluation

**Figure 6:**
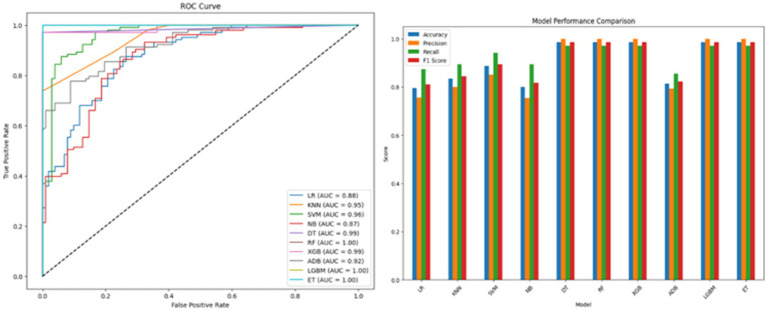
ROC curve and performance comparison of baseline ML models.

In the baseline stage models were trained without feature selection or advanced processing to assess their performance on raw data. ROC AUC ( $\mathrm{RO}C_{\mathrm{AUC}}$ ), $A_{\mathrm{cc}}$ , $P_{\mathrm{rn}}$ , $R_{\mathrm{cl}}$ and $F_{1s}$ are the evaluation metrics. Table 7 summarizes the results while Figure 6 presents the ROC curve and model comparison. DT, RF, XGB, LGBM and ET achieved the highest accuracy of 98.53%. SVM also performed well with an $A_{\mathrm{cc}}$ of 88.78% and a high $\mathrm{RO}C_{\mathrm{AUC}}$ of 0.9631. KNN and ADB demonstrated competitive performance with $\mathrm{RO}C_{\mathrm{AUC}}$ values of 0.9508 and 0.9225, respectively. These results highlight the effectiveness of ensemble methods and SVM as strong baselines though lower accuracy and higher variability indicate the need for feature selection and optimization.

**Table 7:** Baseline ML model performance.

Model	$A_{\mathrm{cc}}(\%)$	$P_{\mathrm{rn}}(\%)$	$R_{\mathrm{cl}}(\%)$	$F_{1s}$	$\mathrm{RO}C_{\mathrm{AUC}}$
LR	79.51	75.63	87.37	0.8108	0.8790
KNN	83.41	80.00	89.32	0.8440	0.9508
SVM	88.78	85.08	94.17	0.8940	0.9631
NB	80.00	75.40	89.32	0.8177	0.8705
**DT**	**98.53**	**100.00**	**97.08**	**0.9852**	**0.9854**
**RF**	**98.53**	**100.00**	**97.08**	**0.9852**	**0.9985**
**XGB**	**98.53**	**100.00**	**97.08**	**0.9852**	**0.9894**
ADB	81.46	79.27	85.43	0.8224	0.9225
**LGBM**	**98.53**	**100.00**	**97.08**	**0.9852**	**1.0000**
**ET**	**98.53**	**100.00**	**97.08**	**0.9852**	**1.0000**

Bold values indicate the jointly best performance within the table.

##### 4.5.1.2 Impact of RST feature selection on ML models

**Figure 7:**
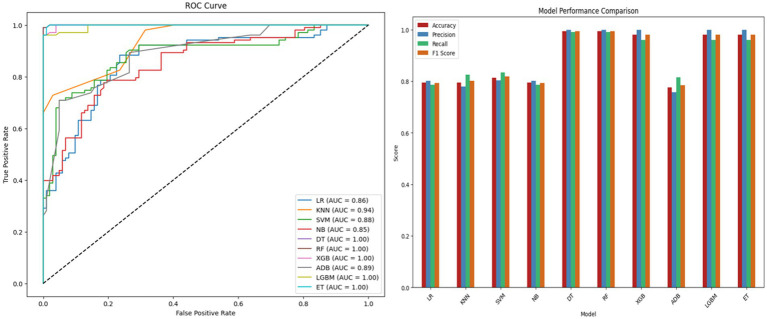
ROC curve and performance comparison of RST-ML model.

Feature selection is carried out using RST which reduces dimensionality by retaining only the most significant features. In turn, this improves model efficiency by eliminating redundancy and irrelevance resulting in improved $A_{\mathrm{cc}}$ , $P_{\mathrm{rn}}$ , $R_{\mathrm{cl}}$ and $F_{1s}$ . The selected features are trained using various ML models to assess performance. The results are summarized in Table 8 with Figure 7 providing a comparative visualization of the ROC curves and model comparison. DT, RF, XGB, LGBM and ET outperformed other models achieving perfect precision, high recall and F1 scores with $\mathrm{RO}C_{\mathrm{AUC}}$ values approaching 1. The SVM model achieved an $A_{\mathrm{cc}}$ of 81.46% and a solid $\mathrm{RO}C_{\mathrm{AUC}}$ of 0.8847. The results indicate that RST effectively improves model efficiency by retaining the most informative features.

**Table 8:** RST-ML model performance.

Model	$A_{\mathrm{cc}}(\%)$	$P_{\mathrm{rn}}(\%)$	$R_{\mathrm{cl}}(\%)$	$F_{1s}$	$\mathrm{RO}C_{\mathrm{AUC}}$
RST + LR	79.51	80.19	78.64	0.7941	0.8648
RST + KNN	79.51	77.98	82.52	0.8018	0.9365
RST + SVM	81.46	80.37	83.49	0.8190	0.8847
RST + NB	79.51	80.19	78.64	0.7941	0.8548
**RST + DT**	**98.15**	**100.00**	**99.02**	**0.9951**	**0.9989**
**RST + RF**	**98.15**	**100.00**	**99.02**	**0.9951**	**0.9989**
**RST + XGB**	98.04	100.00	96.11	0.9801	0.9987
RST + ADB	77.56	75.67	81.55	0.7850	0.8903
**RST + LGBM**	98.04	100.00	96.11	0.9801	0.9955
**RST + ET**	98.04	100.00	96.11	0.9801	0.9995

Bold values indicate the jointly best performance within the table.

##### 4.5.1.3 Effect of RNS representation on RST selected features

Feature selection using RST reduces the original feature set to a small and informative subset improving learning efficiency by removing redundant and irrelevant attributes. To examine whether the observed performance gains arise from uncertainty aware representation rather than increased model complexity an ablation study was conducted by comparing RST selected features with standard normalization against the same features enhanced using RNS under identical classifiers and training conditions. The RNS transformation does not introduce new independent features. Instead, each selected attribute is represented through bounded T, I and F components capturing uncertainty under lower and upper approximations. This study demonstrates that every component is necessary to provide a successful and efficient task specific final model architecture.

Table 9 show consistent performance improvements after RNS integration. DT and RF classifiers improve their accuracy from 98.15 to 99.51% while maintaining near-perfect precision and achieving higher recall and F1-scores. These results indicate that the improvement is driven by enhanced uncertainty representation rather than dimensional expansion. By explicitly modeling ambiguous and overlapping instances RNS improves class separability and leads to more robust predictive performance. This configuration evaluates the contribution of deep feature extraction by OptiDANet by directly training the RF classifier on RNS transformed features without using the OptiDANet architecture.

**Table 9:** RNS enhanced ML model performance.

Model	$A_{\mathrm{cc}}(\%)$	$P_{\mathrm{rn}}(\%)$	$R_{\mathrm{cl}}(\%)$	$F_{1s}$	$\mathrm{RO}C_{\mathrm{AUC}}$
RNS + LR	79.51	80.19	78.64	0.7941	0.8648
RNS + KNN	80.00	78.70	82.52	0.8056	0.9362
RNS + SVM	81.46	80.37	83.49	0.8190	0.8845
RNS + NB	79.51	80.19	78.64	0.7941	0.8548
**RNS + DT**	**98.51**	**99.00**	**98.02**	**0.9851**	**0.9898**
**RNS + RF**	**98.51**	**99.00**	**98.02**	**0.9851**	**0.9898**
RNS + XGB	98.04	100.00	96.11	0.9801	0.9987
RNS + ADB	77.56	75.67	81.55	0.7850	0.8903
RNS + LGBM	98.04	100.00	96.11	0.9801	0.9955
RNS + ET	98.04	100.00	96.11	0.9801	0.9987

Bold values indicate the jointly best performance within the table.

**Figure 8:**
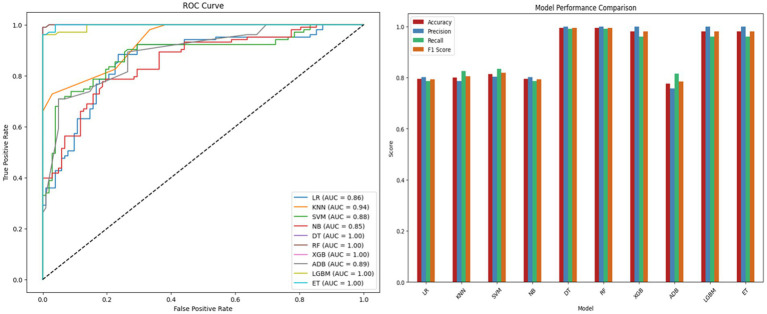
ROC curve and performance comparison of RST-RNS-ML models.

These results highlight the advantages of incorporating RNS representation to handle data imprecision ultimately leading to more robust and reliable models. A detailed performance comparison along with the ROC curve analysis is provided in Figure 8.

##### 4.5.1.4 Impact of CAM and SAM within the RNS-OptiDANet framework

To explicitly evaluate the contribution of the CAM and SAM, the RNS-OptiDANet framework was compared against the RNS based configuration without deep feature extraction. While both settings use identical RST selected features and RNS representations, the RNS-OptiDANet model additionally incorporates CAM and SAM for adaptive feature recalibration and contextual modeling. The performance gains observed in Table 10 over Table 9 therefore directly quantify the impact of the CAM and SAM modules.

**Table 10:** Performance of proposed RNS-OptiDANet approach on ML models.

Model	$A_{\mathrm{cc}}(\%)$	$P_{\mathrm{rn}}(\%)$	$R_{\mathrm{cl}}(\%)$	$F_{1s}(\%)$	$\mathrm{RO}C_{\mathrm{AUC}}$
RNS-OptiDANet + LR	85.85	85.87	85.85	0.8585	0.9231
RNS-OptiDANet + KNN	84.39	84.43	84.39	0.8438	0.9584
RNS-OptiDANet + SVM	86.82	86.85	86.82	0.8682	0.9318
RNS-OptiDANet + NB	81.46	81.50	81.46	0.8145	0.8787
**RNS-OptiDANet + DT**	**99.51**	**99.51**	**99.51**	**0.9951**	**0.9998**
**RNS-OptiDANet + RF**	**99.51**	**99.51**	**99.51**	**0.9951**	**0.9999**
**RNS-OptiDANet + XGB**	**99.51**	**99.51**	**99.51**	**0.9951**	**0.9998**
RNS-OptiDANet + ADB	82.92	82.95	82.92	0.8292	0.9543
**RNS-OptiDANet+LGBM**	**99.51**	**99.51**	**99.51**	**0.9951**	**0.9998**
**RNS-OptiDANet + ET**	**99.51**	**99.51**	**99.51**	**0.9951**	**0.9998**

Bold values indicate the jointly best performance within the table.

All classifiers used the same preprocessing step and identical training-testing split. Each model was trained and evaluated using the selected feature subset obtained from the RST-QRDM approach, followed by RNS transformation. This design ensures that performance differences rise from the learning algorithms and the addition of CAM and SAM, rather than from differences in data preparation.

**Figure 9:**
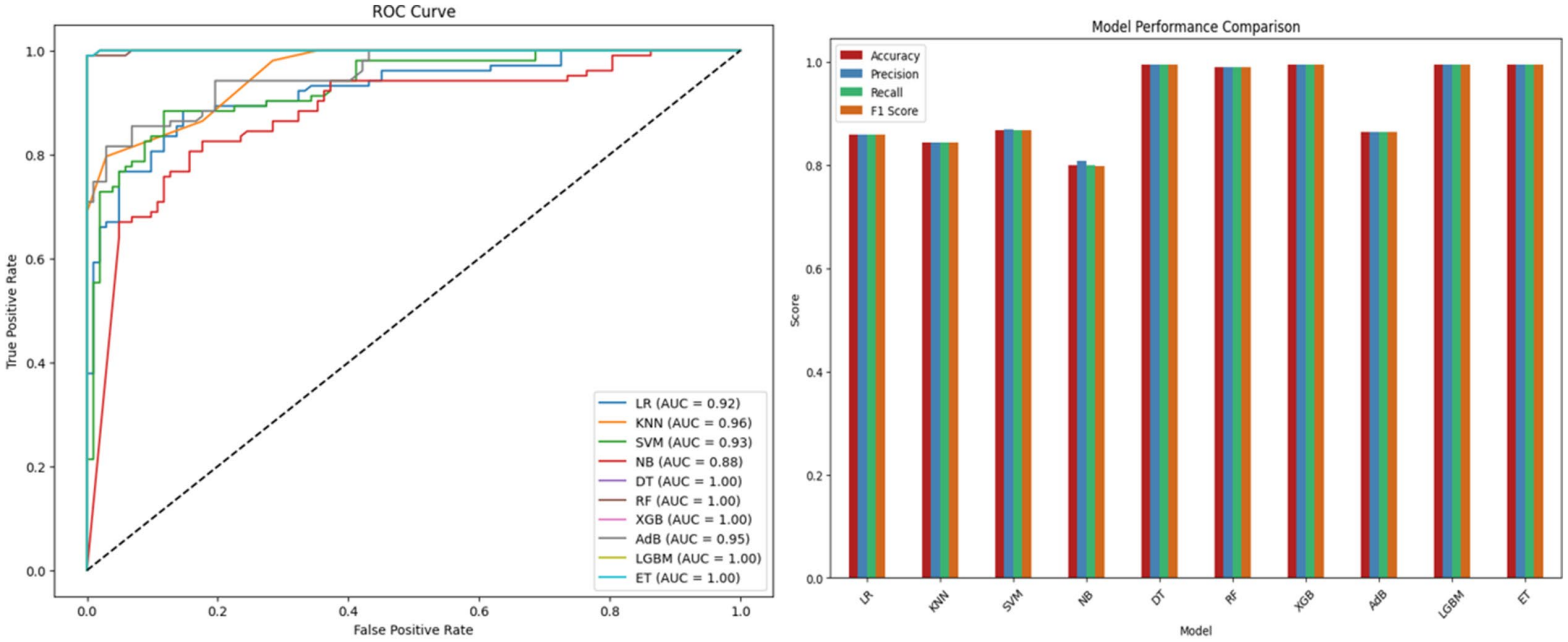
Performance comparison and ROC curve of the proposed RNS-OptiDANet approach.

Table 10 shows that the proposed RNS-OptiDANet approach resulted in a significant increase in classification performance with DT, RF, XGB, LGBM and ET achieving an accuracy of 99.51% and $\mathrm{RO}C_{\mathrm{AUC}}$ values approaching 1. Models such as LR and NB showed improvements with increased accuracy and stable performance across all evaluation metrics. These findings indicate that CAM enhances channel-wise discriminability by highlighting informative feature maps whereas SAM improves spatial or contextual aggregation resulting in strong and more robust representations. Figure 9 depicts the ROC curve and model comparison of the RNS-OptiDANet approach.

For final evaluation, multiple ML classifiers were employed instead of restricting the approach to RF alone. Optuna was used to optimize the OptiDANet feature extraction block which transforms high dimensional RNS data into a refined representation. The extracted features were then classified using different ML models for stable and accurate prediction. This constant optimization strategy ensures fairness while allowing each classifier to perform under its optimal configuration.

The integration of RST based feature selection, RNS representation and attention based deep feature extraction leads to a structurally consistent and performance enhancing process. The observed improvements confirm that the proposed RNS-OptiDANet approach effectively strengthens feature representation.

##### 4.5.1.5 ML Model selection using MCDM-TOPSIS for the proposed RNS-OptiDANet approach

**Figure 10:**
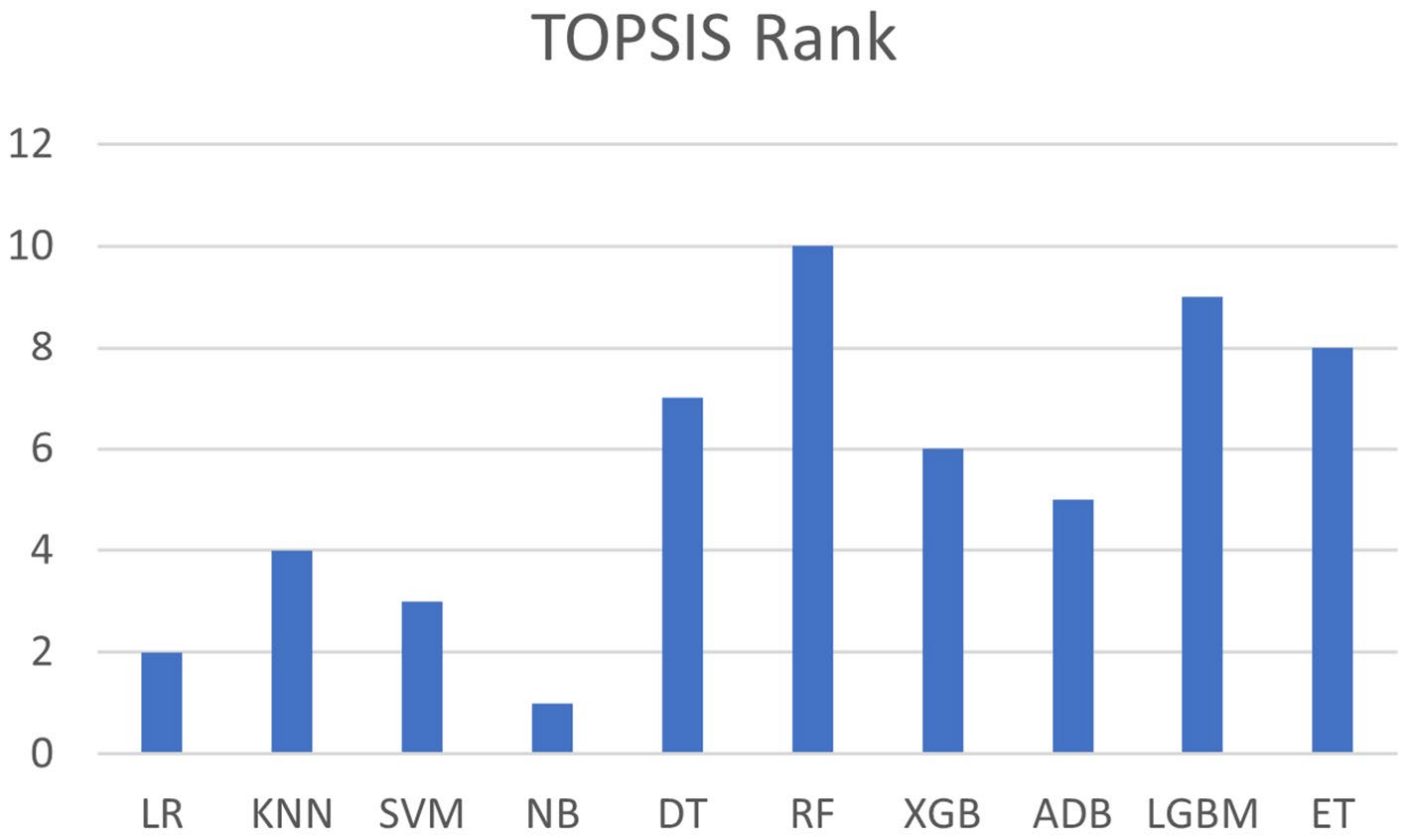
TOPSIS Ranking of Models Based on Performance Scores.

To select the optimal ML model for the proposed RNS-OptiDANet approach, MCDM technique particularly the TOPSIS method is employed to rank the ML model. Table 10, shows that the DT, RF, XGB, LGBM and ET models exhibit similar performance across various metrics. To identify a single optimal model for the proposed RNS-OptiDANet method, TOPSIS ranking is applied to evaluate and select the best performing classifier. Using the MEREC method criteria weights were determined. The model rankings based on the TOPSIS performance scores are presented in Figure 10.

It is clear from Figure 10 that the RF model ranks first. Therefore, RF is selected as the final model for the proposed RNS-OptiDANet approach.

##### 4.5.1.6 Summary comparison across Variants of the Proposed RNS-OptiDANet approach

A comparison is presented in Table 11 to improve clarity among the different experimental setups of the proposed RNS-OptiDANet approach. As demonstrated by the TOPSIS ranking the RF classifier consistently achieves the highest performance score when multiple models exhibit comparable metric values. Therefore, RF is selected as the representative model for each framework variant in Table 11. The results indicate a clear and progressive improvement as the framework evolves from baseline ML to the proposed RNS-OptiDANet approach confirming the increasing benefit of RST based feature selection, RNS representation and OptiDANet based feature refinement.

**Table 11:** Best Model Comparison Across Framework Configurations.

Framework	Model	$A_{\mathrm{cc}}(\%)$	$P_{\mathrm{rn}}(\%)$	$R_{\mathrm{cl}}(\%)$	$F_{1s}(\%)$	$\mathrm{RO}C_{\mathrm{AUC}}$
Baseline ML	RF	98.53	100.00	97.08	0.9852	0.9985
RST-ML	RF	98.15	100.00	99.02	0.9951	0.9989
RNS-ML	RF	98.51	99.00	98.02	0.9851	0.9898
**RNS-OptiDANet**	**RF**	**99.51**	**99.51**	**99.51**	**0.9951**	**0.9999**

Bold values indicate the best performance within the table.

##### 4.5.1.7 Discussion on the component-wise ablation analysis

This research presents a comprehensive evaluation of various ML models with progressive performance enhancements achieved through the integration of RST, RNS and the OptiDANet model. The baseline result highlighted the superior performance of ensemble models such as RF, XGB and LGBM which demonstrated high accuracy and near-perfect precision. The application of RST for feature selection effectively improved model efficiency by reducing dimensionality while retaining critical information. The integration of RNS facilitated enhanced uncertainty management leading to further improvements in classification performance.

The final integrated RNS-OptiDANet model combining RST, RNS and OptiDANet yielded near-optimal classification results significantly outstanding other approaches. To identify a single effective model within the proposed RNS-OptiDANet approach, TOPSIS method was employed. RF was identified as the final model by the TOPSIS analysis highlighting its capacity to successfully capture complex data patterns. Based on these findings RNS-OptiDANet model was proposed as the optimal model.

The ablation results confirm that the observed performance improvements are not solely due to RNS representation or classifier choice but are significantly influenced by the addition of CAM and SAM within the OptiDANet feature extractor.

#### 4.5.2 Ablation analysis on feature cardinality

To further analyze the influence of feature subset size on classification performance, an ablation study was conducted by varying the number of selected features within the proposed framework. Using the RST based feature selection mechanism feature subsets with cardinalities k = 3, k = 7 and k = 10 were evaluated and compared against the proposed configuration with k = 5 features.

For all configurations the selected features were first transformed using the RNS representation to effectively model uncertainty and indeterminacy in the data. The transformed features were then processed through the dual attention network for feature refinement followed by classification using a RF classifier. Model performance was evaluated on a common test set using $A_{\mathrm{cc}}$ , $P_{\mathrm{rn}}$ , $R_{\mathrm{cl}}$ and $F_{1s}$ . The comparative performance across different feature cardinalities is reported in Table 12.

**Table 12:** Performance comparison across feature cardinalities.

Number of features	$A_{\mathrm{cc}}(\%)$	$P_{\mathrm{rn}}(\%)$	$R_{\mathrm{cl}}(\%)$	$F_{1s}(\%)$
**k = 3**	70.24	0.7040	0.7022	0.7017
**k = 5 (proposed)**	**99.51**	**0.9952**	**0.9952**	**0.9951**
**k = 7**	86.83	0.8713	0.8681	0.8680
**k = 10**	95.61	0.9573	0.9560	0.9561

Bold values indicate the best performance within the table.

##### 4.5.2.1 Performance with k = 3 features

**Figure 11:**
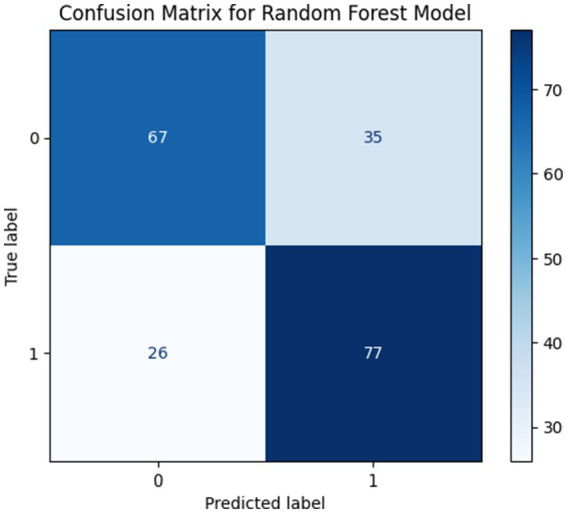
Confusion matrices for k = 3 Selected Features.

When the feature cardinality was reduced to k = 3 the model achieved an accuracy of 70.24% with precision, recall and F1-score of 0.7040, 0.7022 and 0.7017, respectively. The significant performance degradation indicates that excessive feature reduction leads to the loss of critical discriminative information. As a result the model exhibits limited capability in distinguishing between closely related classes. The confusion matrix shown in Figure 11 indicates significant misclassification across multiple performance categories for k = 3 selected features.

##### 4.5.2.2 Performance with k = 7 features

**Figure 12:**
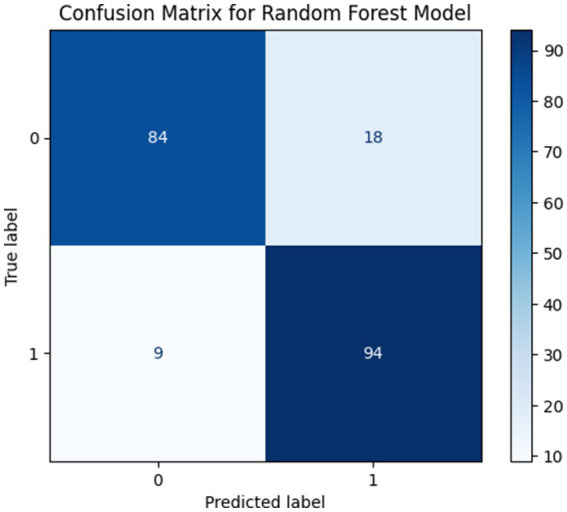
Confusion matrices for Performance with k = 7 Features.

When the number of selected features was increased to k = 7 the model showed an accuracy of 86.83% and precision, recall and F1-score of 0.8713, 0.8681 and 0.8680, respectively. Although the inclusion of additional features enhanced class separability compared to the k = 3 configuration the model still exhibited misclassifications. This indicates that while increasing the number of features can enhance performance. Figure 12 presents the confusion matrix corresponding to the model performance with k = 7 selected features.

##### 4.5.2.3 Performance with k = 10 features

**Figure 13:**
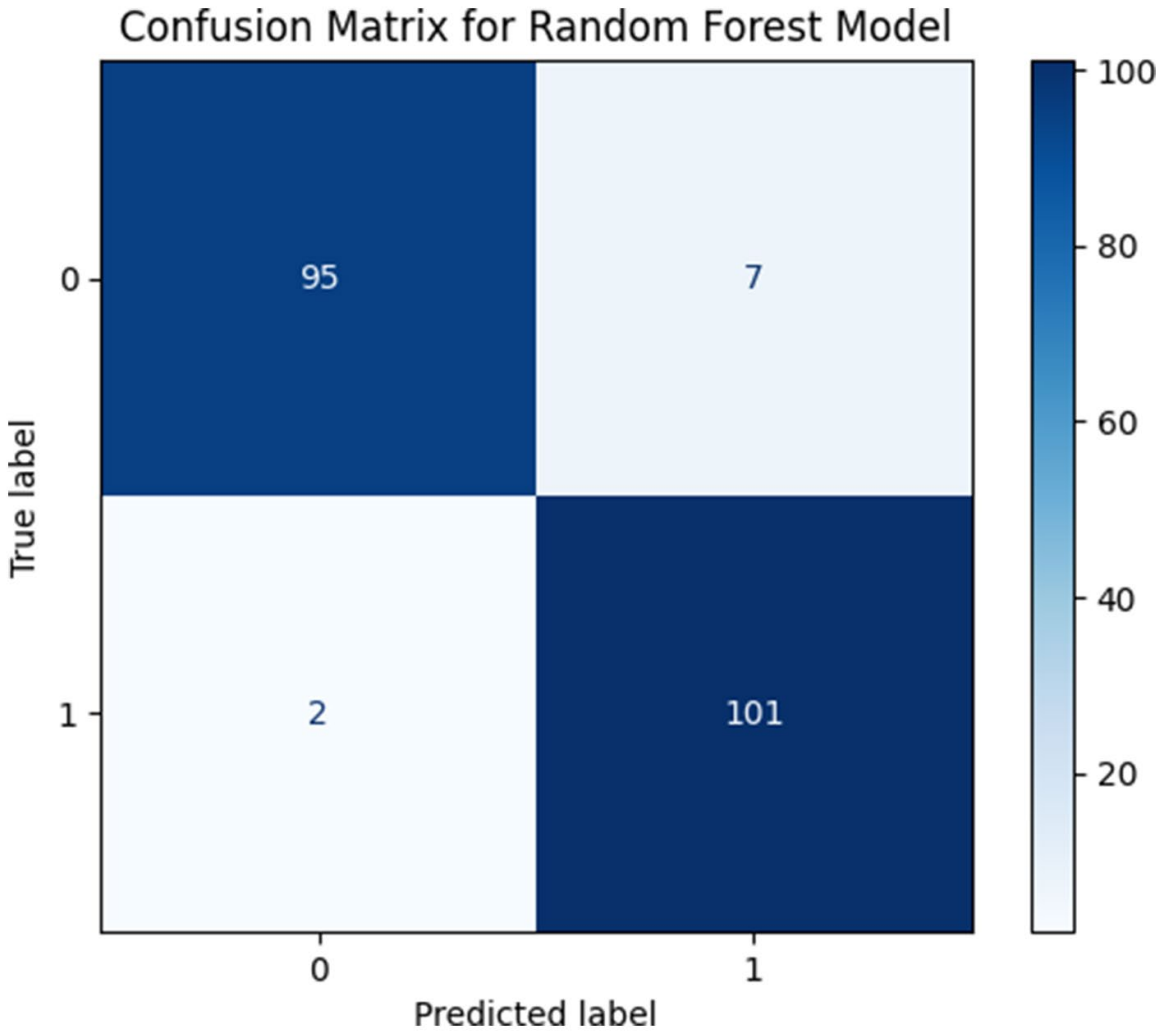
Confusion matrices for Performance with k = 10 Features.

With k = 10 selected features the model achieved a high accuracy of 95.61%. The results demonstrate that increasing feature cardinality improves predictive performance but the performance gains remain inferior to the proposed k = 5 configuration. This observation indicates that incorporating a larger number of features may introduce redundancy leading to limited improvements and an increased risk of overfitting. The confusion matrix for this configuration is presented in Figure 13.

##### 4.5.2.4 Optimal feature cardinality with k = 5

The proposed configuration with k = 5 selected features achieved the best overall performance with an accuracy of 99.51% and precision, recall and F1-score all exceeding 0.995. This result demonstrates that the selected five features provide an effective balance between information sufficiency and model generalization eliminating redundant attributes while preserving the most discriminative features. The corresponding confusion matrix in Figure 5 shows strong diagonal dominance with minimal misclassification.

##### 4.5.2.5 Attention mechanisms

To justify the architectural design of OptiDANet a component wise ablation study was conducted by selectively enabling CAM, SAM, both or no attention. Table 13 shows that CAM alone improves performance over the baseline RF by recalibrating feature importance while SAM alone leads to substantial degradation due to unstable feature interactions. When CAM and SAM are jointly applied the model achieves the highest accuracy of 99.51% confirming that CAM stabilizes feature representations before SAM captures inter-feature dependencies. This demonstrates that the proposed dual-attention design in OptiDANet architecture is complementary rather than redundant.

**Table 13:** Component-wise ablation of attention mechanisms.

Model variant	$A_{\mathrm{cc}}(\%)$	$P_{\mathrm{rn}}(\%)$	$R_{\mathrm{cl}}(\%)$	$F_{1s}(\%)$
No Attention	98.51	0.9900	0.9802	0.9851
SAM	54.63	0.5713	0.5526	0.5176
CAM	98.54	0.9854	0.9857	0.9854
**CAM + SAM (OptiDANet)**	**99.51**	**0.9952**	**0.9952**	**0.9951**

Bold values indicate the best performance within the table.

##### 4.5.2.6 Statistical significance analysis

McNemar’s test was applied to evaluate the statistical significance of the observed performance differences by comparing the proposed k = 5 configuration with alternative feature subsets (k = 3, 7, and 10) using paired predictions on the same test set. The results in Table 14 indicates that the performance improvements achieved by the k = 5 configuration are statistically significant in all cases. These findings confirm that the superior performance of the proposed feature cardinality is not due to random variation.

**Table 14:** McNemar’s test results.

Comparison	McNemar χ2	*p*-value	Significance
k = 5 vs. k = 3	615.3	< 0.0001	Significant
k = 5 vs. k = 7	264.8	< 0.0001	Significant
k = 5 vs. k = 10	81.0	< 0.0001	Significant

### 4.6 Evaluation of existing methods for heart disease prediction

A comparative analysis of existing ML models for heart disease prediction is presented in Table 15 outlining their methodologies and contributions in feature selection and classification. The proposed RNS-OptiDANet model outperforms the compared methods with a high classification $A_{\mathrm{cc}}$ of 99.51%, enhancing feature representation.

**Table 15:** Comparative analysis of ML models for heart disease prediction.

Model/method	Model category	Key approach	Key contributions	$A_{\mathrm{cc}}$ (%)
XGB & RF with SHAP analysis (El-Sofany et al., 2024)	Ensemble & XAI	Hybrid feature selection with XAI	Identifies key risk factors, supports early detection	97.57
GAPSO-RF (El-Shafiey et al., 2022)	Imbalanced aware ensemble	GA + PSO optimized RF	Enhances RF performance using bio-inspired feature selection	95.6 & 91.4
CSRS model (Acharjya, 2020)	Uncertainty aware	Cuckoo Search + RST	Efficient decision rule generation with reduced rule complexity	93.7
CNN with SCSO Feature Selection (Lenin & Venkatasalam, 2025)	Deep learning	CNN with Sand Cat Swarm Optimization (SCSO) for feature selection; PCA, RBM and GAN-based feature extraction	Combines swarm optimization with deep learning to improve ECG-based heart disease classification and prognosis	99.0
Dual attention model for WSI (Raza et al., 2024)	Attention based deep learning	Soft (SAM) and hard attention mechanisms	Highlights relevant regions, reduces computation by ~75%	-
Optimized RST-ML framework (Ashika and Hannah Grace, 2025)	Stacked ensemble & XAI	RST feature selection, ensemble classifiers, MCDM ranking	High accuracy with interpretable stacked models across multiple diseases	98.70
**RNS-OptiDANet (proposed)**	Uncertainty aware attention-based hybrid model	RST-QRDM for feature selection, RNS representation, OptiDANet feature refinement	Robust uncertainty modeling, deep feature extraction, and high generalization performance	**99.51**

**Note:** Bold values indicate the best performance within the table.

While transformer based and end-to-end DL models have shown strong performance in medical prediction, they require large datasets and high computational power. In contrast the proposed RNS-OptiDANet framework is designed for structured clinical highlighting emphasizing uncertainty modeling, interpretability and computational efficiency. The comparison in Table 15 therefore focuses on relevent ensemble, attention-based and uncertainty aware models that are most relevant to tabular medical prediction tasks.

### 4.7 Cross-Dataset Validation of the Proposed RNS-OptiDANet Approach

To evaluate external generalization capability of the proposed RNS-OptiDANet model was further tested on independent open-source datasets collected from different populations and clinical environments. This evaluation aimed to examine its effectiveness in diverse related disease prediction tasks including the Obesity Levels dataset, the Breast Cancer Wisconsin Diagnostic (WDBC) dataset and the CKD dataset. These datasets were selected for their varied characteristics to provide a comprehensive assessment of the model’s robustness and adaptability.

The Wang and Ma (2009) contains 2,111 records from Colombia, Peru and Mexico classified into seven obesity categories based on 17 features related to diet and physical activity. It includes categorical, binary and continuous variables with 77% of records generated via SMOTE and 23% collected from online users, making it a complex multiclass classification task. The WDBC dataset (Breiman, 2001) consists of 569 instances and 30 real valued features extracted from breast mass images used to distinguish benign from malignant tumors. Since the dataset contains no missing values, it provides a clean and reliable benchmark for evaluating classification performance.

The Ekundayo (2020) consists of 400 records with 25 clinical features including blood cell counts and blood pressure, with the target indicating CKD status. During preprocessing, records with missing values were removed during preprocessing. The proposed model’s performance across these datasets was evaluated using key metrics with results presented in Table 16.

**Table 16:** Evaluation of the proposed method on multiple datasets.

Datasets	Obesity levels	Breast cancer	CKD
$A_{\mathrm{cc}}(\%)$	97.48	98.83	98.75
$P_{\mathrm{rn}}(\%)$	97.57	98.41	99.54
$R_{\mathrm{cl}}(\%)$	97.61	98.41	96.67
$F_{1s}$	0.9762	0.9841	0.9831

**Figure 14:**
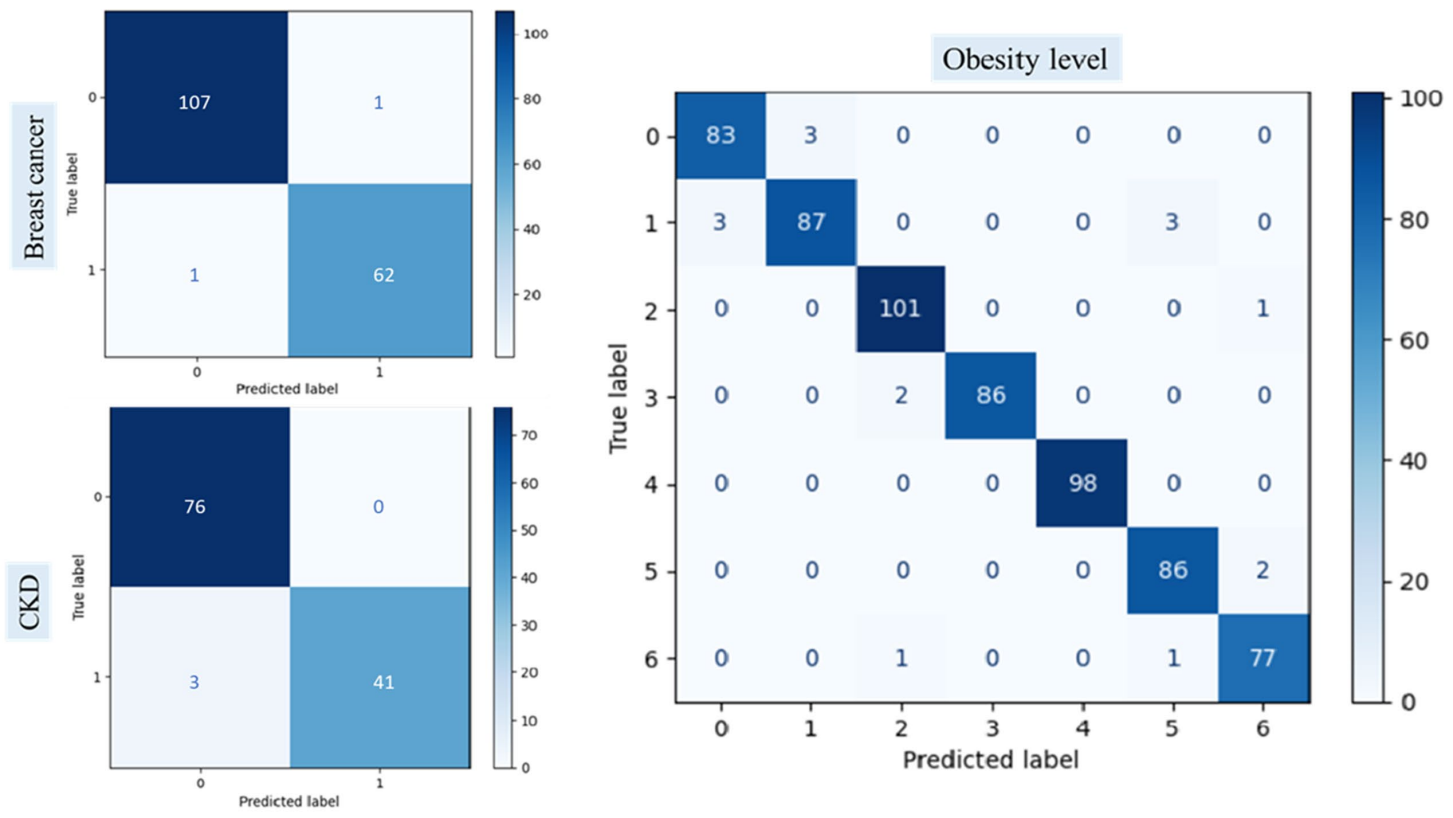
Confusion matrix of the diverse dataset for the proposed RNS-OptiDANet model.

Table 16 highlights strong performance of the RNS-OptiDANet model’s strong performance across multiple datasets. The model achieved an $A_{\mathrm{cc}}$ of 97.48% on the Obesity Levels dataset, 98.83% on the Breast Cancer dataset and 98.75% on the CKD dataset demonstrating robustness in handling for both binary and multiclass classification tasks. These results highlight the model’s effectiveness in healthcare related applications and its potential for real-world disease prediction. The corresponding confusion matrices are presented in Figure 14.

The improved generalization capability of the proposed RNS-OptiDANet framework can be attributed to several key design choices. The RNS representation explicitly models uncertainty, indeterminacy and boundary regions in the data reducing sensitivity to noise and dataset-specific variations. The integration of CAM and SAM enables adaptive feature recalibration allowing the model to focus on informative patterns while suppressing irrelevant or redundant features across different datasets. Optuna based hyperparameter optimization helps prevent overfitting by systematically identifying stable and well performing training configurations rather than relying on dataset specific manual tuning. The hybrid learning strategy which combines deep feature extraction with a RF classifier enhances robustness by leveraging ensemble decision boundaries. These factors collectively contribute to consistent performance of the proposed RNS-OptiDANet model across heterogeneous datasets as confirmed by the multi dataset validation and ablation results.

### 4.8 Interpretation of the proposed RNS-OptiDANet using SHAP and LIME

**Figure 15:**
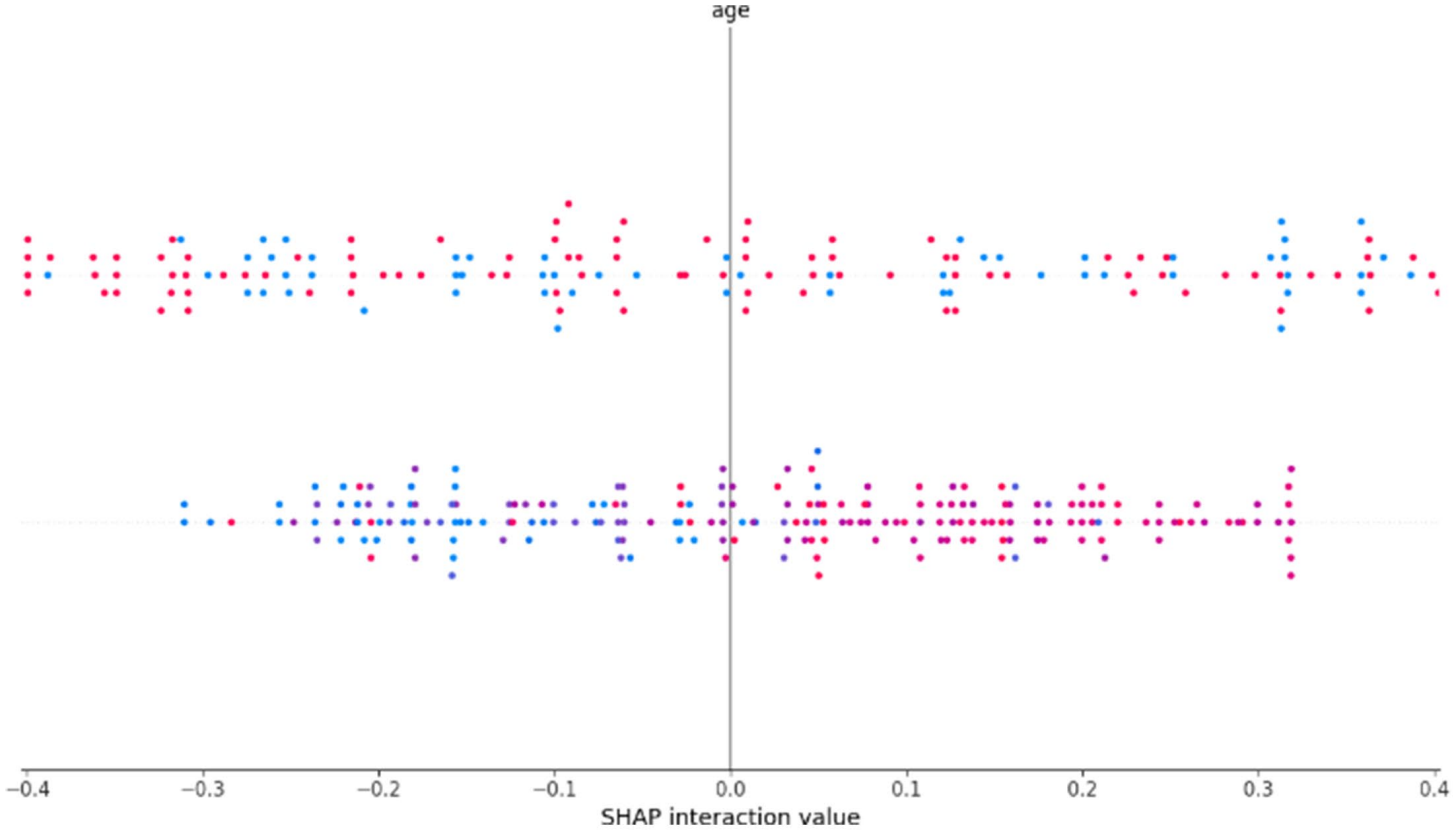
SHAP global feature importance plot for the RNS-OptiDANet model.

To enhance clinical interpretability, the proposed RNS-OptiDANet framework was analyzed using SHAP and LIME explanations applied to the final RF classifier trained on the attention refined features. The SHAP global feature importance plot shown in Figure 15 highlights cp as the most influential feature exhibiting a strong positive contribution toward heart disease prediction followed by trestbps and fbs. Features such as age and gender demonstrate comparatively moderate yet consistent effects which align with established clinical knowledge.

**Figure 16:**
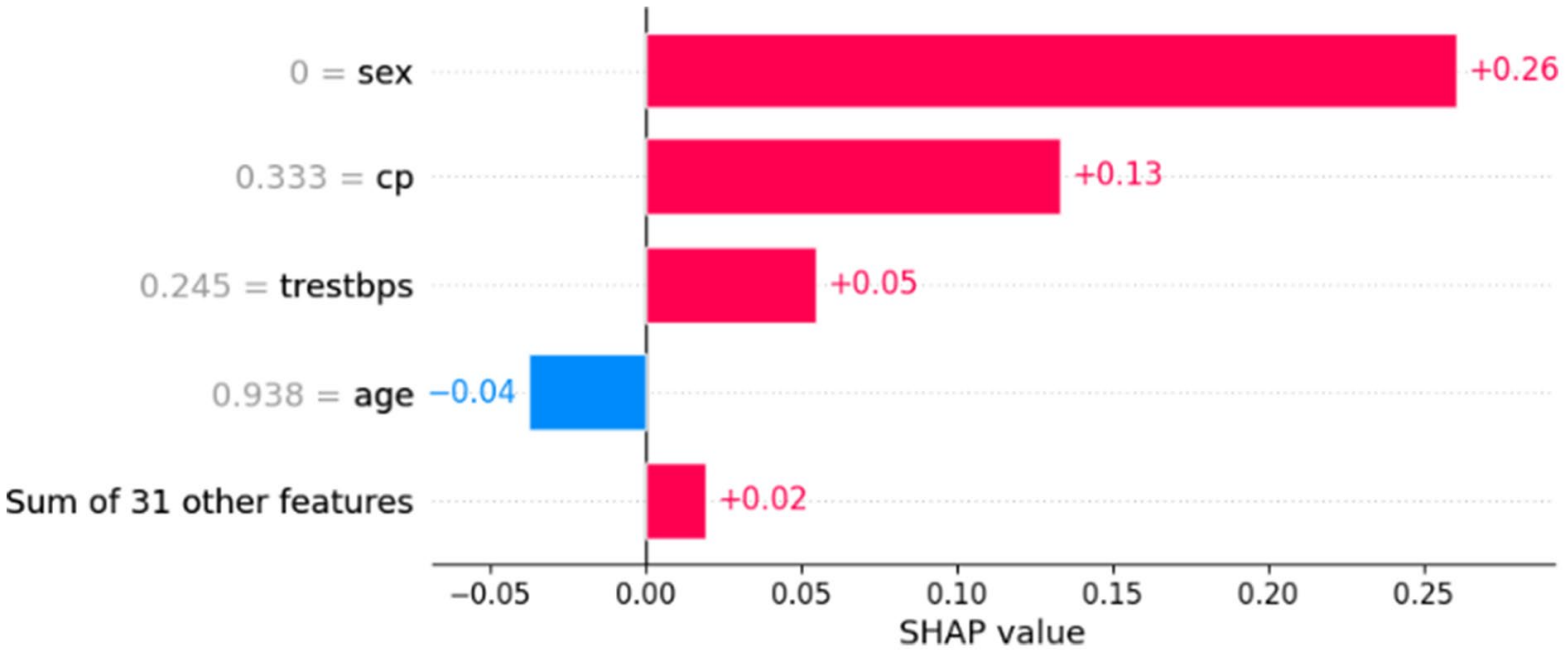
SHAP summary and interaction plot illustrating feature interactions in the RNS-OptiDANet model.

The SHAP summary and interaction analysis presented in Figure 16 further reveals nuanced interactions involving age. Higher age values exhibit both positive and negative interaction effects with other features indicating its complex and context-dependent role in CVD risk assessment.

**Figure 17:**
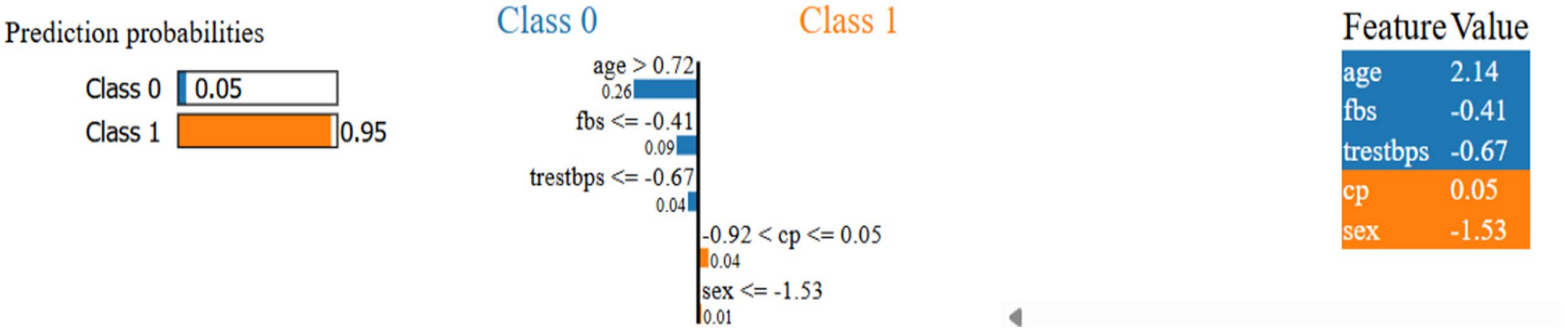
LIME-based local explanation of an individual heart disease prediction using the RNS-OptiDANet model.

**Figure 18:**
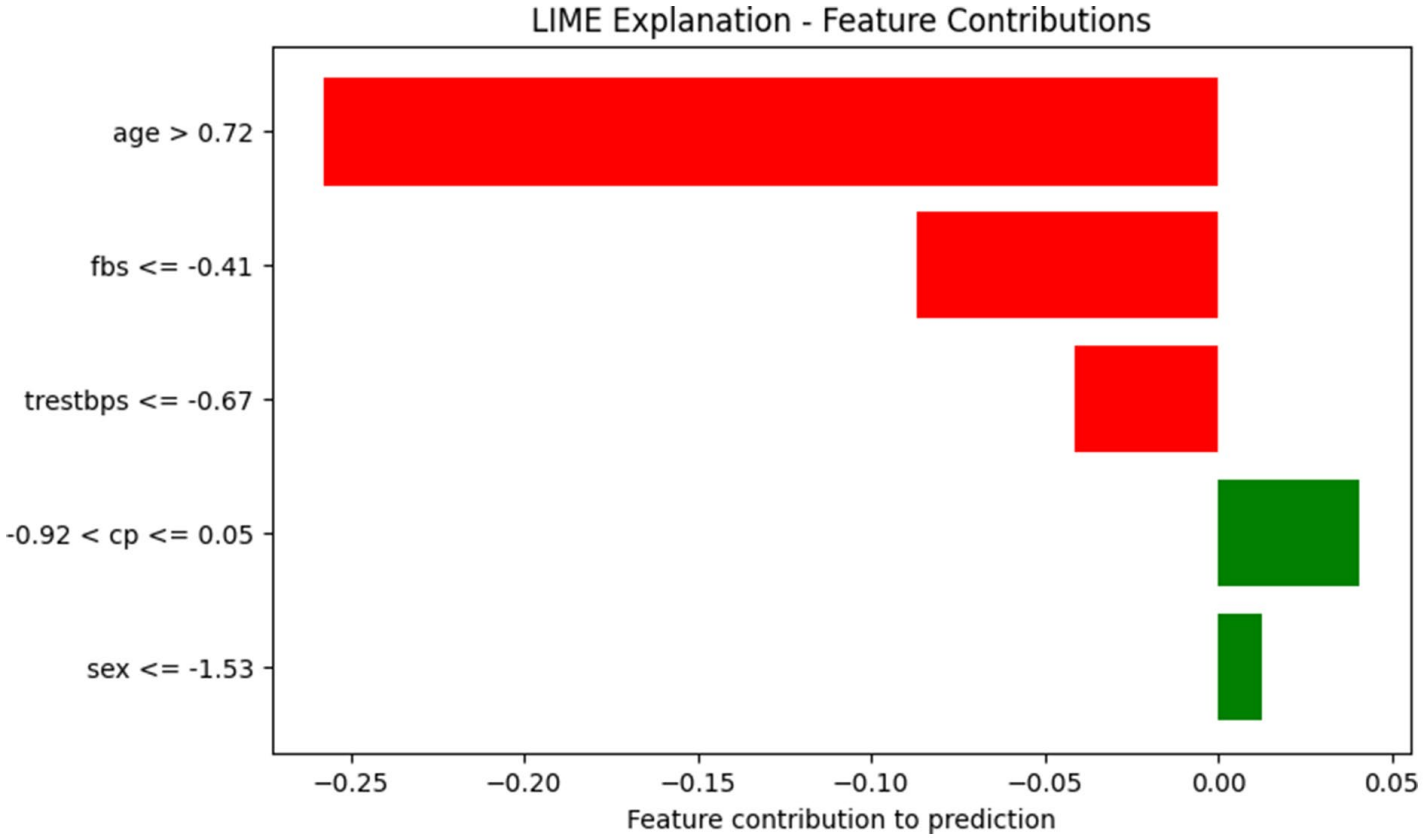
LIME feature contribution bar plot highlighting factors influencing the model’s prediction.

LIME was employed to provide local explanations for individual predictions. Figure 17 illustrates that cp emerges as the dominant positive contributor supporting heart disease prediction while gender, age, trestbps and fbs negatively influence the decision with varying magnitudes. The corresponding LIME bar plot shown in Figure 18 reinforces the predominance of cp while also illustrating how other clinical attributes modulate the final prediction outcome.

Overall, these explainability results demonstrate that the proposed RNS-OptiDANet framework bases its predictions on clinically meaningful factors thereby enhancing transparency and supporting its potential applicability in real world clinical decision making.

### 4.9 Discussion and limitation

The proposed RNS-OptiDANet framework clearly establishes OptiDANet as a feature extraction and refinement module rather than an end-to-end classifier. The dual-attention components such as CAM and SAM enhance channel discrimination and contextual dependency learning producing refined representations that improve downstream classification. Final prediction is performed using a RF classifier which is selected due to its robustness on structured tabular data, strong generalization ability and inherent interpretability. The ablation analysis confirms that performance gains from RNS to RNS-OptiDANet results from improved feature representation rather than increased classifier complexity supporting the justification for the hybrid DL-ML design.

Overfitting control is strengthened through multiple procedures. RST based feature selection minimizes redundancy and reduces variance while the RNS transformation enhances uncertainty aware expressiveness without introducing irrelevant noise. Hyperparameter optimization using Optuna is confined strictly to the training phase using a fixed 70:30 split. Consistent performance across different feature cardinalities along with statistically results from significant McNemar’s test demonstrate that the observed improvements reflect robust generalization rather than optimization bias.

Although the dual-attention mechanism introduces additional training complexity, this cost occurs offline and remains moderate. Once trained, inference with the RNS-OptiDANet model combined with RF is computationally efficient enabling real time or batch clinical screening. The ablation study further validates the architectural choices where CAM improves channel level reliability, SAM alone exhibits inconsistent behavior and their combined application ensures stable and consistent performance gains. These findings validates the necessity of dual attention for robust feature refinement.

Unlike studies that rely on manually tuned hyperparameters, this work employs systematic Optuna based optimization for every model. This reduces human bias and ensures that each classifier is evaluated under its best performing configuration.

While the proposed model requires slightly longer training time due to RNS expansion and attention mechanisms, the increase is manageable. The inference time remains very low which is less than one millisecond per sample making the model practical and suitable for real world clinical deployment.

Alternative approaches such as end-to-end deep classifiers, transformer based models and autoencoder based architectures were not prioritized because the primary objective of this work is uncertainty aware representation combined with interpretable ensemble learning. The focus remains on balancing predictive strength, transparency and structural robustness rather than increasing architectural depth.

Overall, the enhanced discussion clarifies that the observed improvements arise from uncertainty modeling and attention based feature refinement not from classifier complexity or hyperparameter tuning alone.

## 5 Conclusion and future work

The proposed RNS-OptiDANet model achieved 99.51% accuracy in heart disease prediction by integrating RST based feature selection, RNS representation and OptiDANet feature extraction with Optuna optimized hyperparameters. The attention mechanisms embedded in OptiDANet specifically CAM and SAM played a crucial role in enhancing feature discrimination and contextual modeling leading to consistent performance gains across all evaluated classifiers. When trained with an RF classifier, the model demonstrated strong performance across key metrics and maintained high accuracy on diverse datasets, achieving 97.48% for Obesity Levels, 98.83% for Breast Cancer and 98.75% for CKD dataset highlighting its effectiveness in both binary and multiclass medical classification.

Future work will focus on further evaluating the model’s scalability and generalizability by testing it on clinical and more diverse datasets. DL or hybrid models will be explored alongside assessing the real time deployment of the model in clinical settings to further enhance its robustness and applicability in real world environments. Future research could also aim at improving computational efficiency, supporting real time deployment in clinical environments and validating the framework on more external heart disease datasets to strengthen its applicability in real world risk prediction.

## Data Availability

The dataset analysed in this study is publicly available in the UCI Machine Learning Repository: Janosi, A., Steinbrunn, W., Pfisterer, M., and Detrano, R. (1989). Heart Disease Dataset. https://doi.org/10.24432/C52P4X (Accessed 05 March 2026).
